# Supplementary figures and images for: Vulnerable connectivity caused by local communities in spatial networks
Source: PLoS One. 2025 Jul 2;20(7):e0327203. doi: 10.1371/journal.pone.0327203 (PMC12221043; doi:10.1371/journal.pone.0327203)

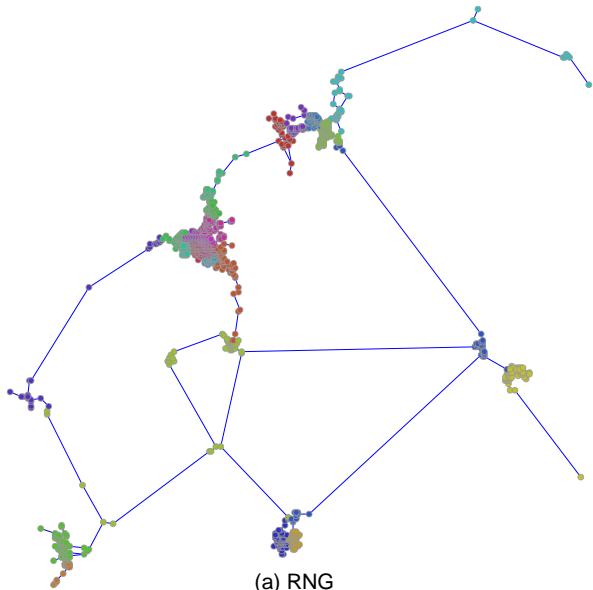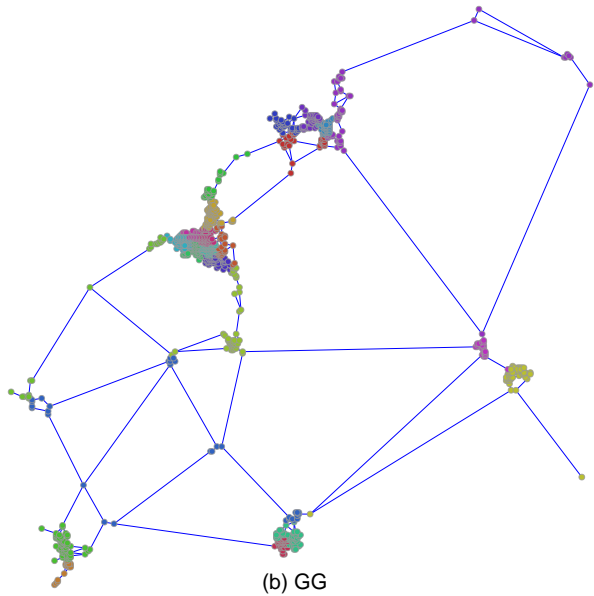

Supplement: S1 Fig — Visualization of community structures in Fukuoka before node removal. N=1024 nodes are located by the decreasing order of population (Pop.). Different colors represent different communities estimated by Louvain method. There are clear community formations particularly in densely populated areas. (PDF) [file pone.0327203.s001.pdf]

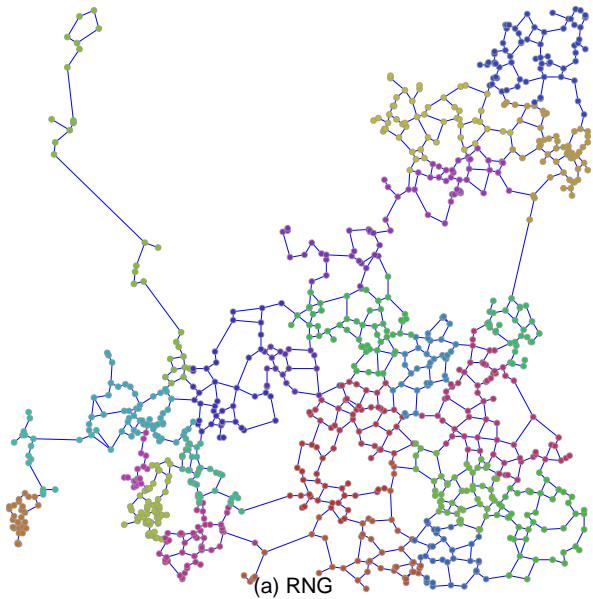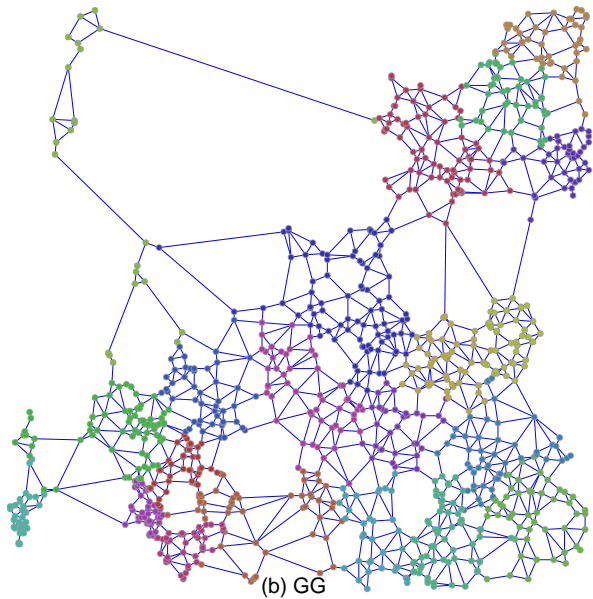

Supplement: S2 Fig — Visualization of community structures in Fukuoka before node removal. N = 1024 nodes are located by the inverse order of population (Inv.). Different colors represent different communities estimated by Louvain method. There are different community formations compared to S1 Fig. (PDF) [file pone.0327203.s002.pdf]

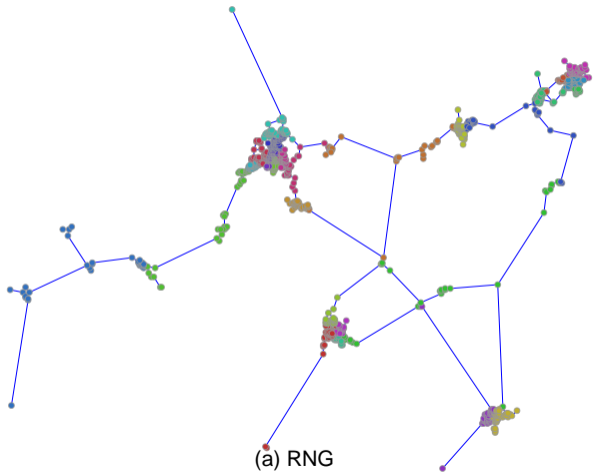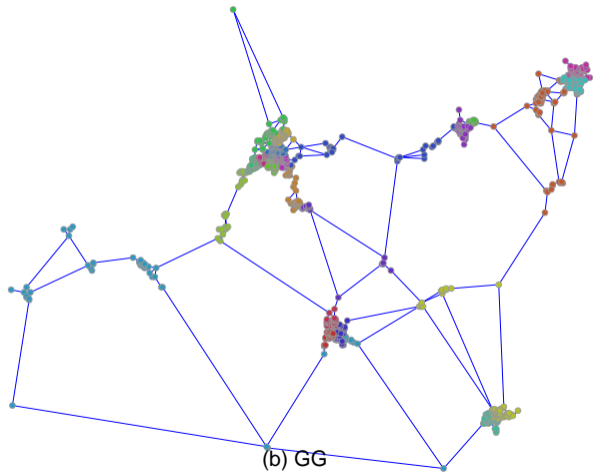

Supplement: S3 Fig — Visualization of community structures in Hiroshima before node removal. N = 1024 nodes are located by the decreasing order of population (Pop.). Different colors represent different communities estimated by Louvain method. There are clear community formations particularly in densely populated areas. (PDF) [file pone.0327203.s003.pdf]

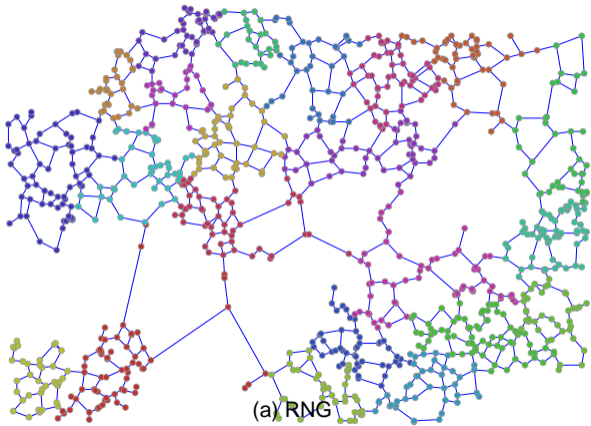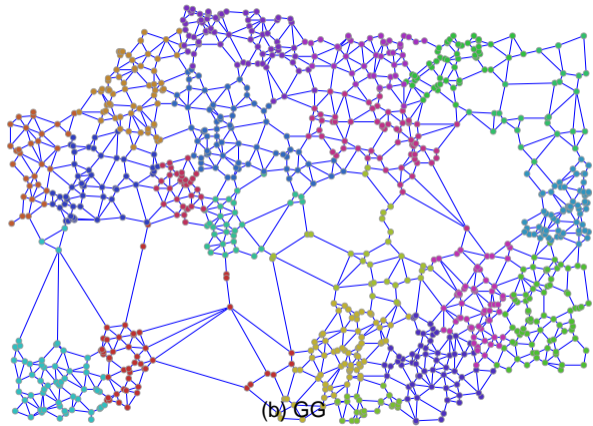

Supplement: S4 Fig — Visualization of community structures in Hiroshima before node removal. N = 1024 nodes are located by the inverse order of population (Inv.). Different colors represent different communities estimated by Louvain method. There are different community formations compared to S3 Fig. (PDF) [file pone.0327203.s004.pdf]

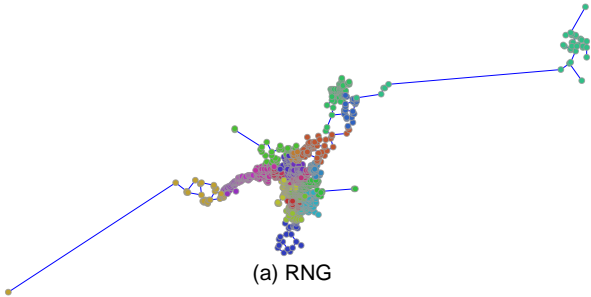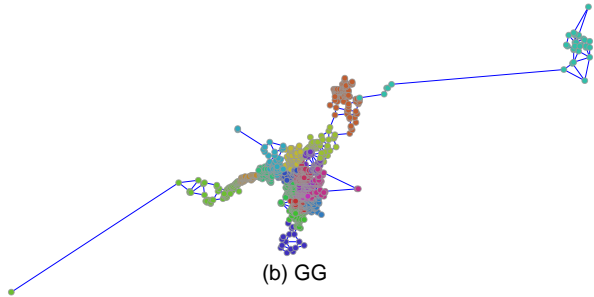

Supplement: S5 Fig — Visualization of community structures in Keihan before node removal. N = 1024 nodes are located by the decreasing order of population (Pop.). Different colors represent different communities estimated by Louvain method. There are clear community formations particularly in densely populated areas. (PDF) [file pone.0327203.s005.pdf]

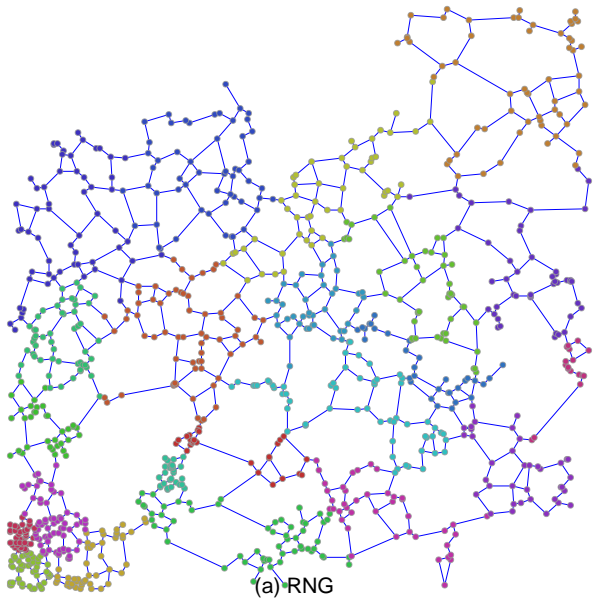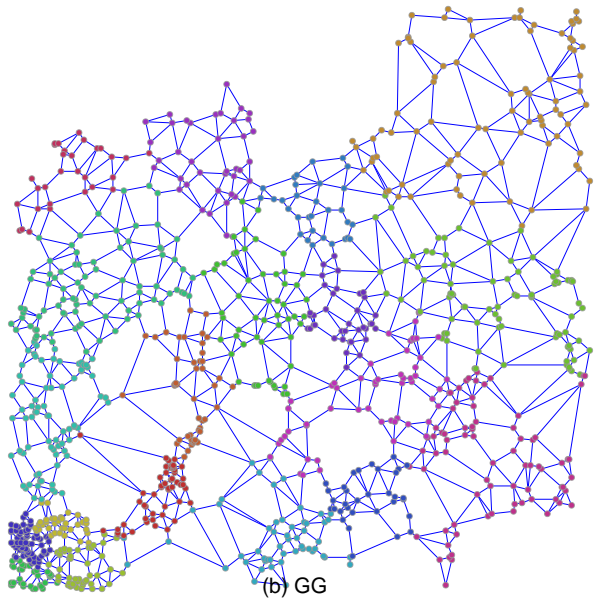

Supplement: S6 Fig — Visualization of community structures in Keihan before node removal. N = 1024 nodes are located by the inverse order of population (Inv.). Different colors represent different communities estimated by Louvain method. There are different community formations compared to S5 Fig. (PDF) [file pone.0327203.s006.pdf]

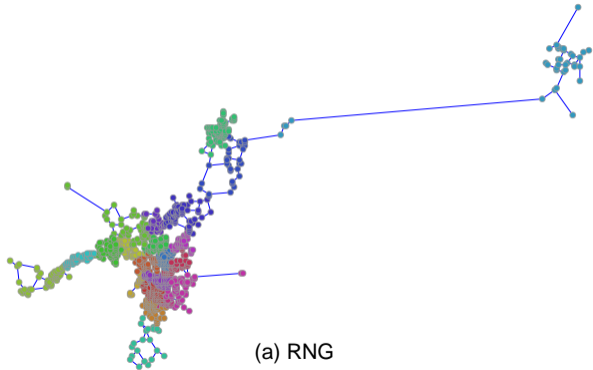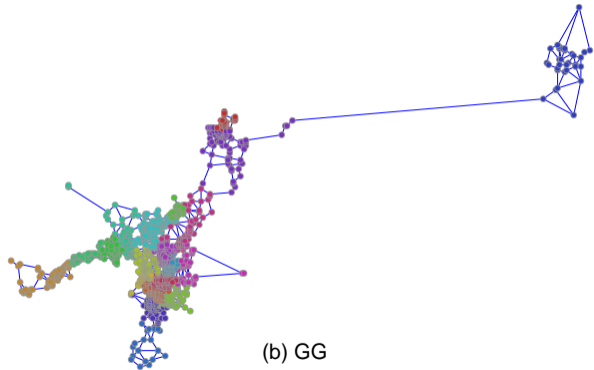

Supplement: S7 Fig — Visualization of community structures in Nagoya before node removal. N = 1024 nodes are located by the decreasing order of population (Pop.). Different colors represent different communities estimated by Louvain method. There are clear community formations particularly in densely populated areas. (PDF) [file pone.0327203.s007.pdf]

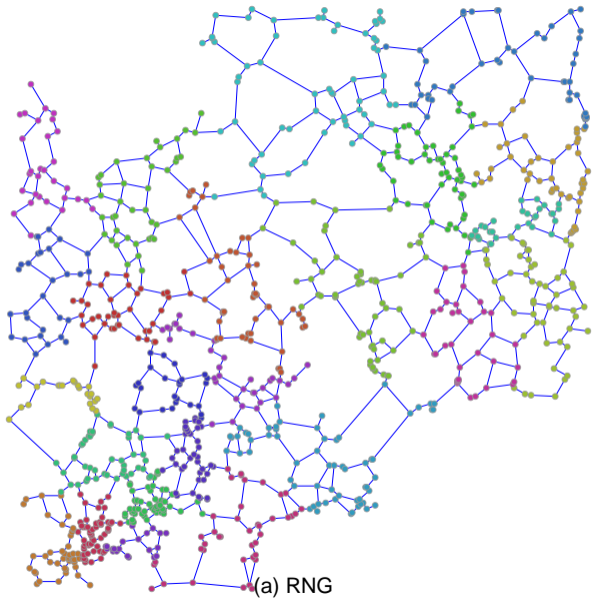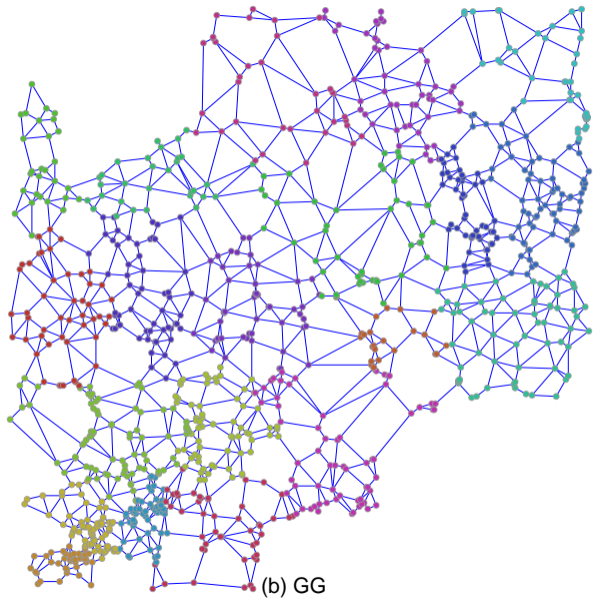

Supplement: S8 Fig — Visualization of community structures in Nagoya before node removal. N = 1024 nodes are located by the inverse order of population (Inv.). Different colors represent different communities estimated by Louvain method. There are different community formations compared to S7 Fig. (PDF) [file pone.0327203.s008.pdf]

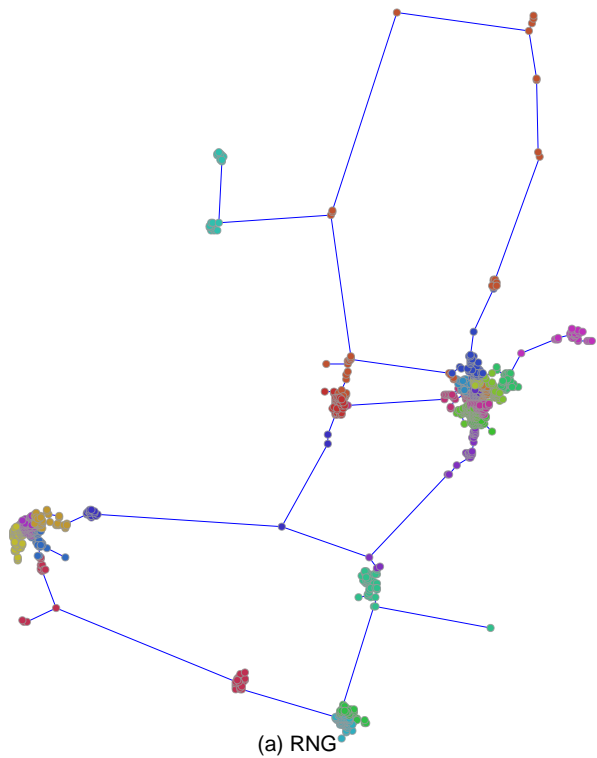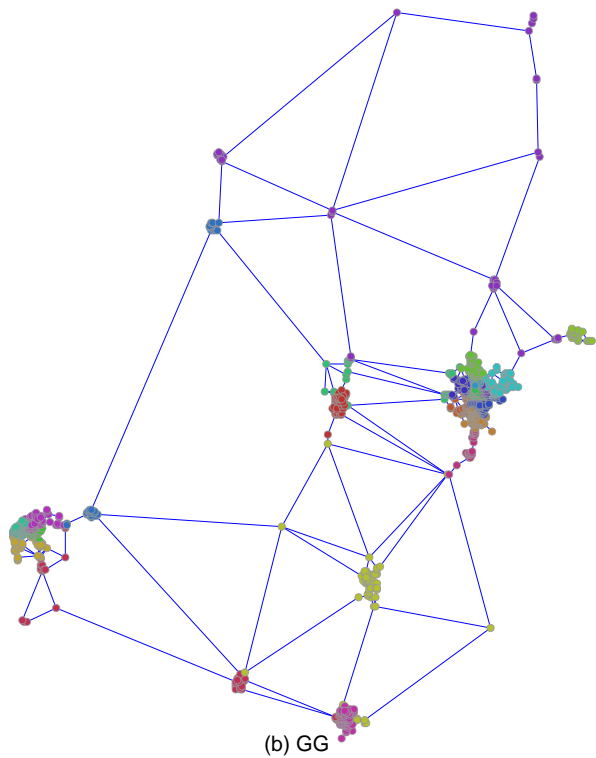

Supplement: S9 Fig — Visualization of community structures in Sendai before node removal. N = 1024 nodes are located by the decreasing order of population (Pop.). Different colors represent different communities estimated by Louvain method. There are clear community formations particularly in densely populated areas. (PDF) [file pone.0327203.s009.pdf]

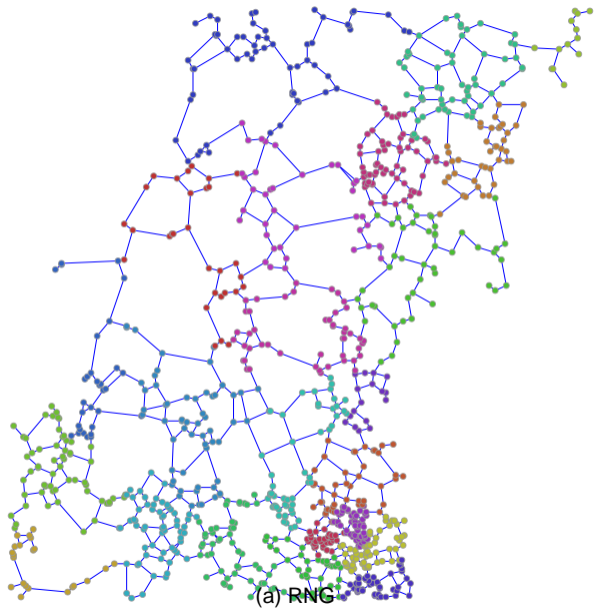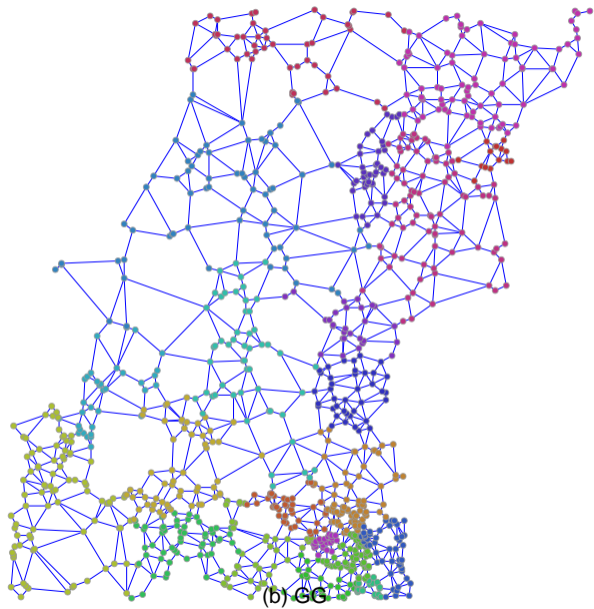

Supplement: S10 Fig — Visualization of community structures in Sendai before node removal. N = 1024 nodes are located by the inverse order of population (Inv.). Different colors represent different communities estimated by Louvain method. There are different community formations compared to S9 Fig. (PDF) [file pone.0327203.s010.pdf]

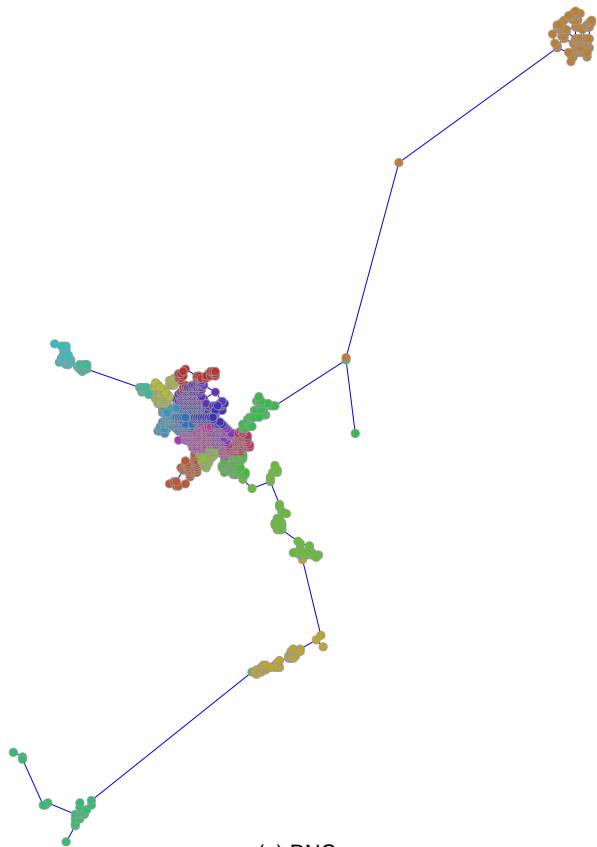

(a) RNG

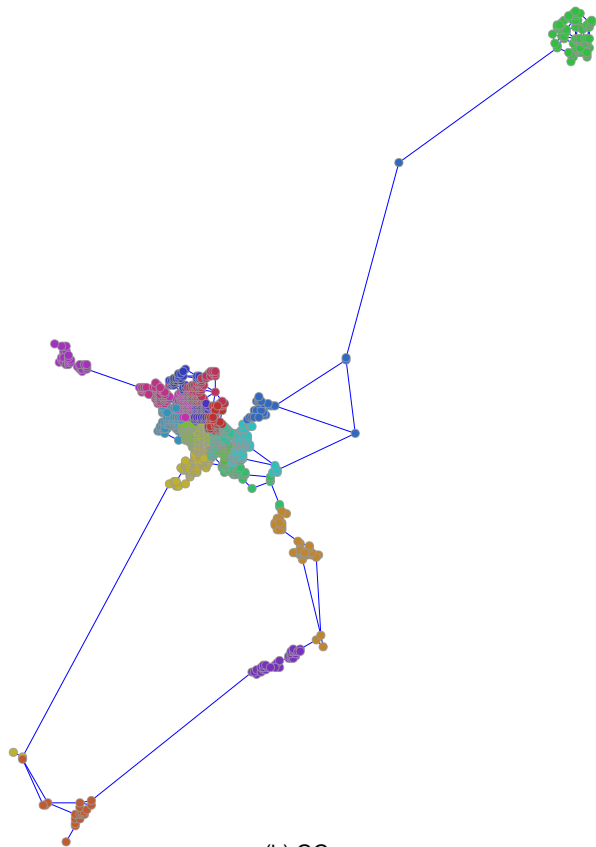

(b) GG

Supplement: S11 Fig — Visualization of community structures in Sapporo before node removal. N = 1024 nodes are located by the decreasing order of population (Pop.). Different colors represent different communities estimated by Louvain method. There are clear community formations particularly in densely populated areas. (PDF) [file pone.0327203.s011.pdf]

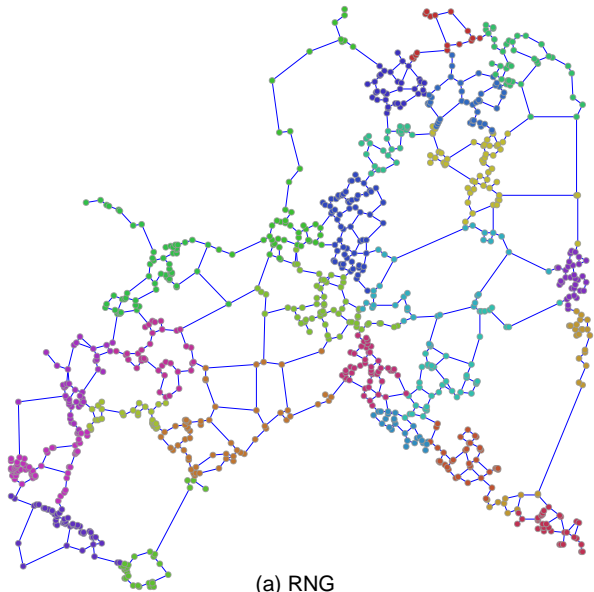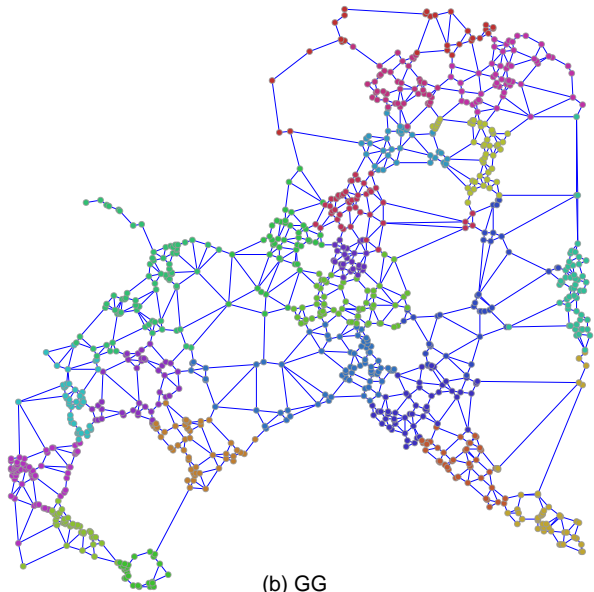

Supplement: S12 Fig — Visualization of community structures in Sapporo before node removal. N = 1024 nodes are located by the inverse order of population (Inv.). Different colors represent different communities estimated by Louvain method. There are different community formations compared to S11 Fig. (PDF) [file pone.0327203.s012.pdf]

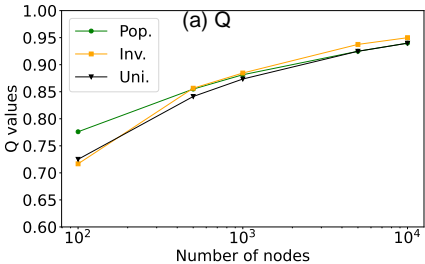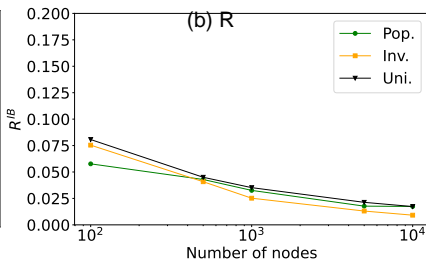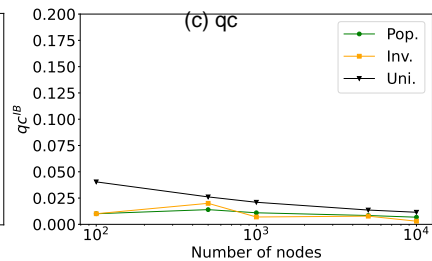

Supplement: S13 Fig — Increasing modularity Q vs. decreasing robustness index RRB or critical fraction qcPRB for varying the size N in Tokyo RNG networks. (PDF) [file pone.0327203.s013.pdf]

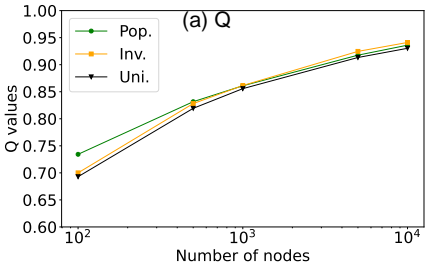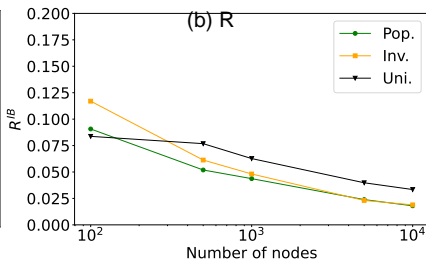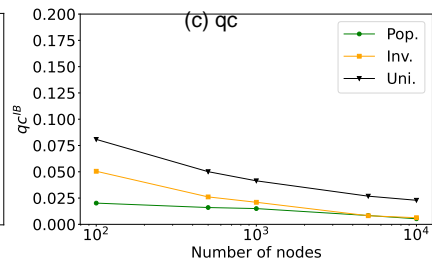

Supplement: S14 Fig — Increasing modularity Q vs. decreasing robustness index RRB or critical fraction qcPRB for varying the size N in Tokyo GG networks. (PDF) [file pone.0327203.s014.pdf]

(a) R

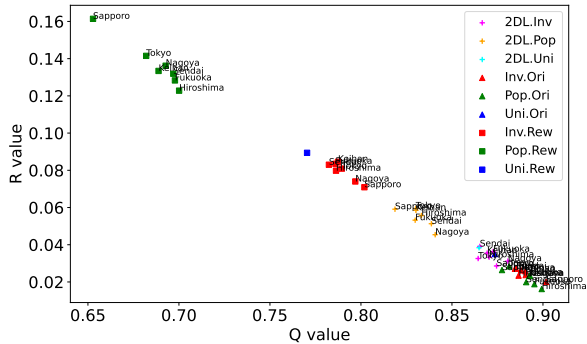

(b) qc

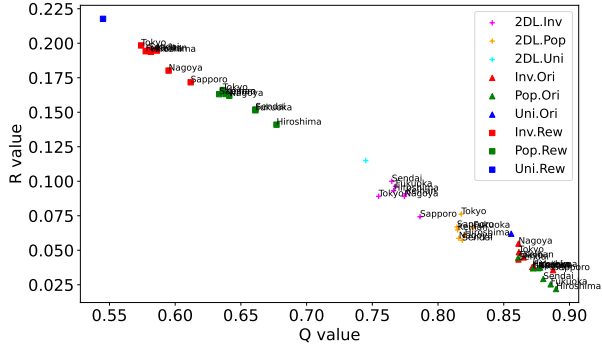

Supplement: S15 Fig — Scatter plots show relation between robustness measures (a for R and b for qc) and the proportion of grid-like parts against random failures (RF). Networks with N = 1024 nodes are considered, where Pop. networks (green) show notably higher proportions of grid-like parts compared to Inv. (red) and Uni. (blue) networks. See the text at the end of subsection 3.3 for the detail. (PDF) [file pone.0327203.s015.pdf]

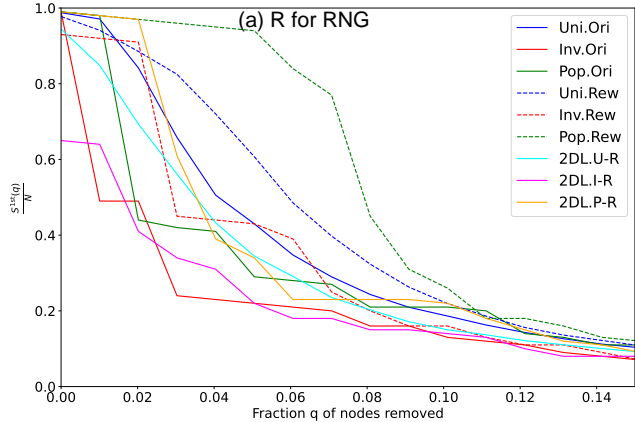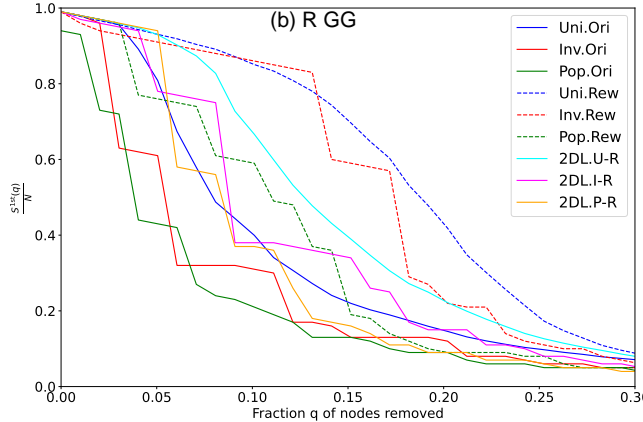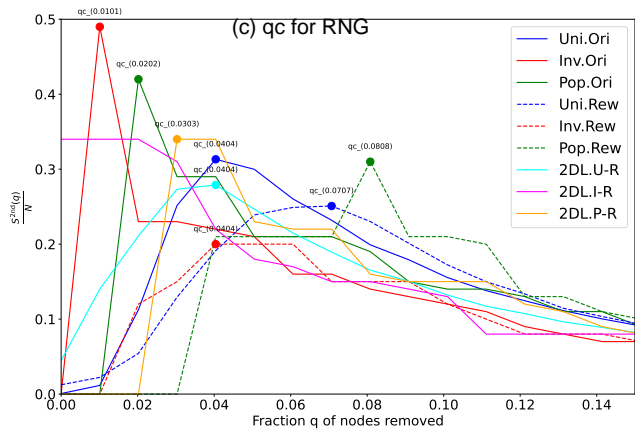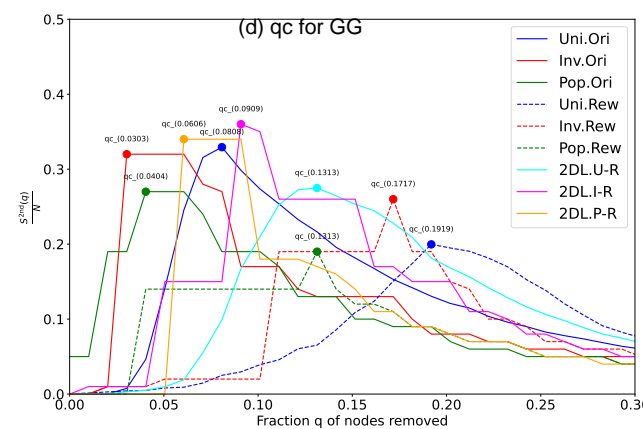

Supplement: S16 Fig — Robustness against recalculated betweenness (RB) attacks for Fukuoka networks with N = 100 nodes. For both Rew (Randomized networks) and 2DL lines, the rewiring process preserves the original degree distributions. Two measures are applied: (a) (b) S1st(q)/N the relative size of largest connected component, and (c) (d) S2nd(q)/N the critical fraction qc at the peak of the relative size of second largest component. (PDF) [file pone.0327203.s016.pdf]

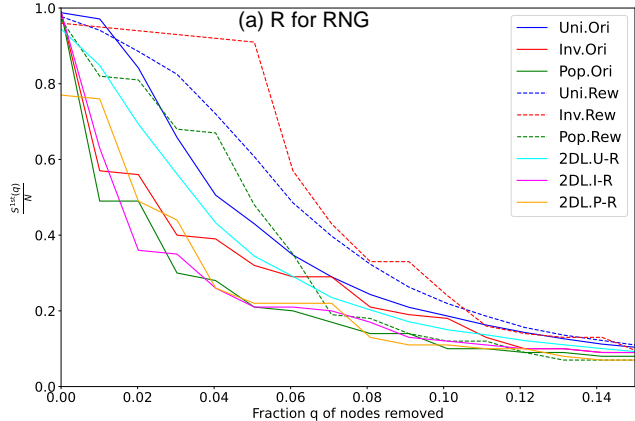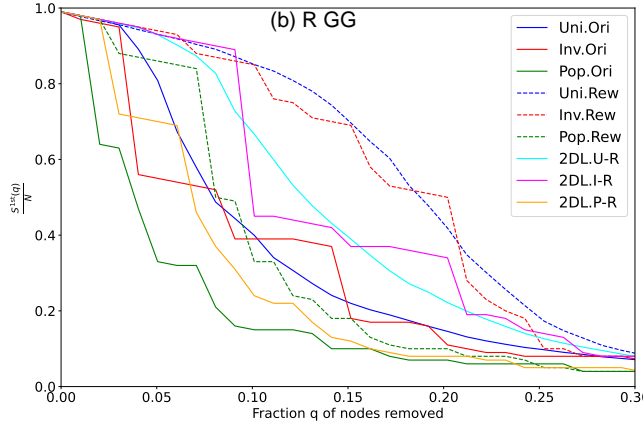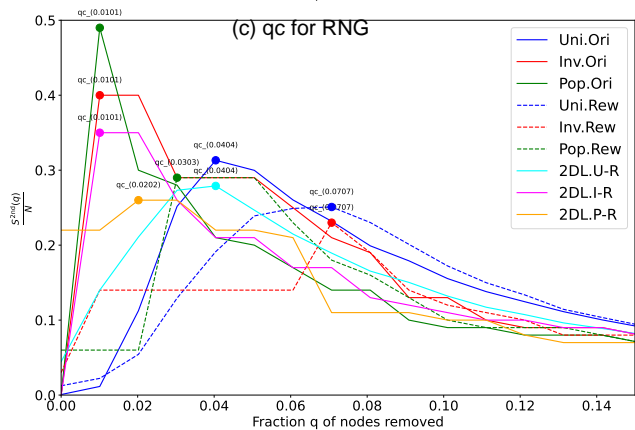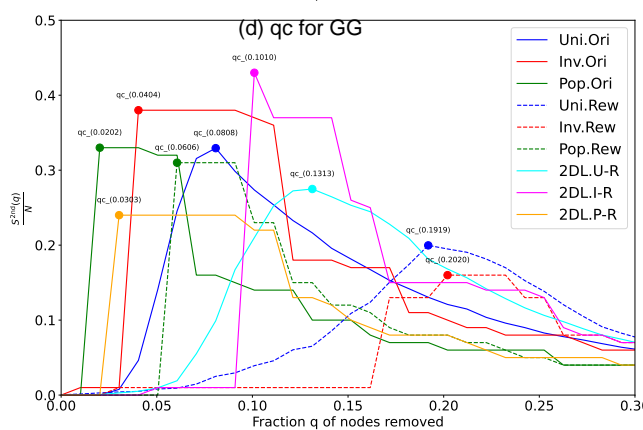

Supplement: S17 Fig — Robustness against recalculated betweenness (RB) attacks for Hiroshima networks with N = 100 nodes. For both Rew (Randomized networks) and 2DL lines, the rewiring process preserves the original degree distributions. Two measures are applied: (a) (b) S1st(q)/N the relative size of largest connected component, and (c) (d) S2nd(q)/N the critical fraction qc at the peak of the relative size of second largest component. (PDF) [file pone.0327203.s017.pdf]

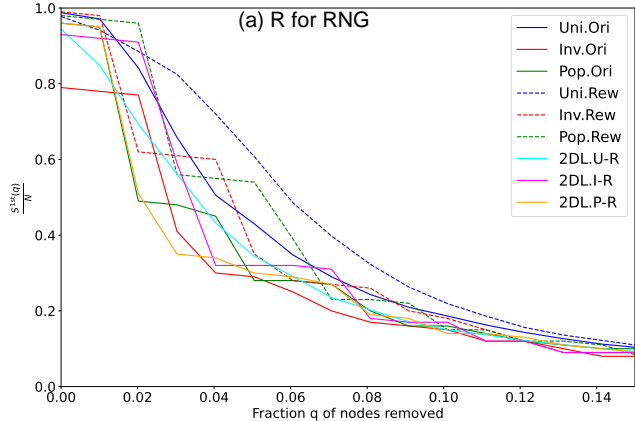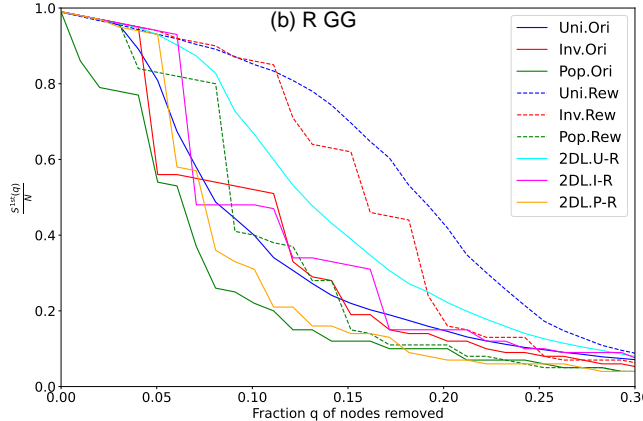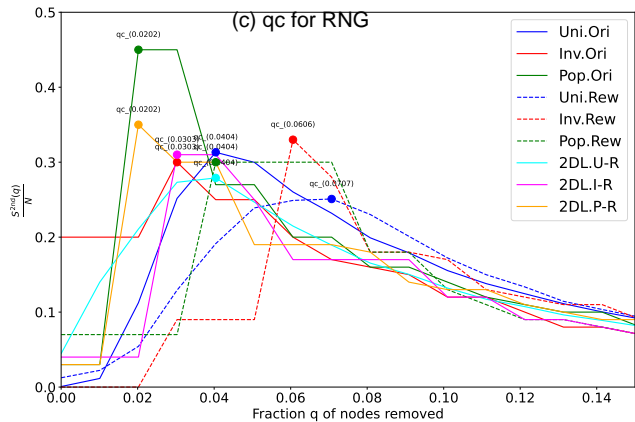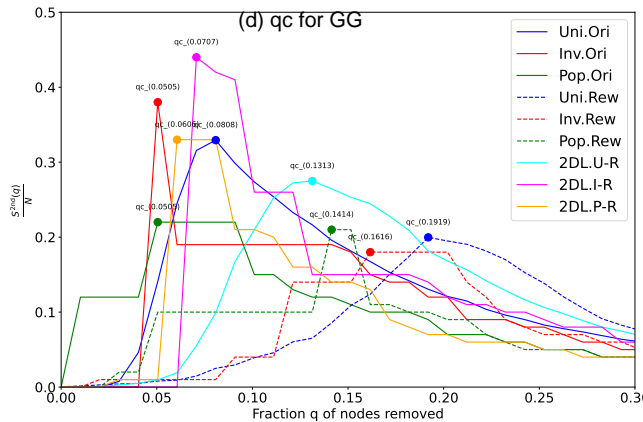

Supplement: S18 Fig — Robustness against recalculated betweenness (RB) attacks for Keihan networks with N = 100 nodes. For both Rew (Randomized networks) and 2DL lines, the rewiring process preserves the original degree distributions. Two measures are applied: (a) (b) S1st(q)/N the relative size of largest connected component, and (c) (d) S2nd(q)/N the critical fraction qc at the peak of the relative size of second largest component. (PDF) [file pone.0327203.s018.pdf]

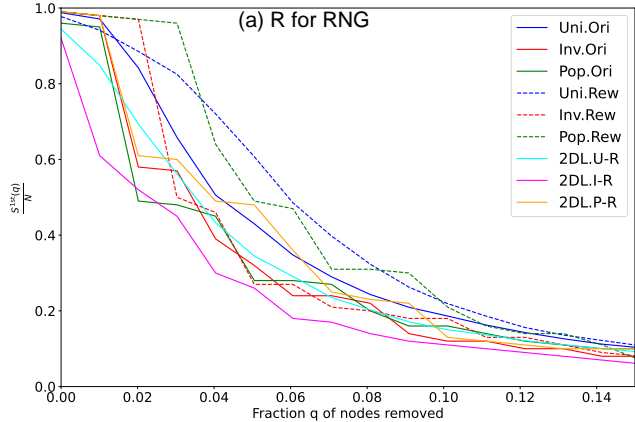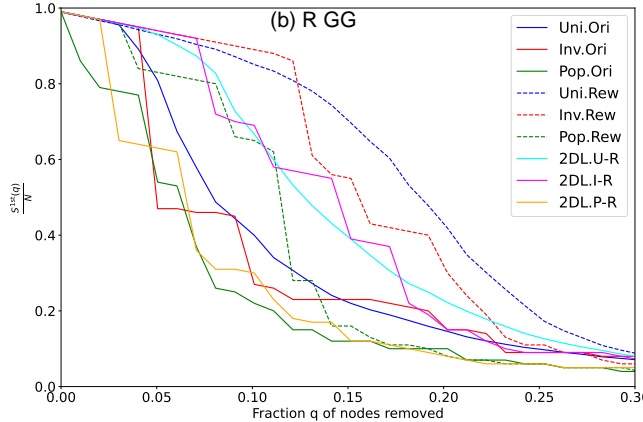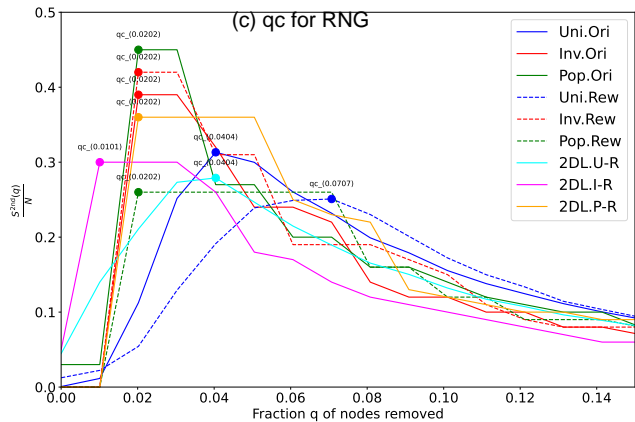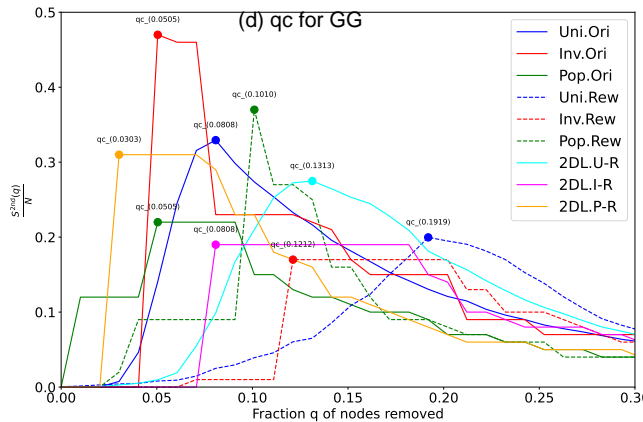

Supplement: S19 Fig — Robustness against recalculated betweenness (RB) attacks for Nagoya networks with N = 100 nodes. For both Rew (Randomized networks) and 2DL lines, the rewiring process preserves the original degree distributions. Two measures are applied: (a) (b) S1st(q)/N the relative size of largest connected component, and (c) (d) S2nd(q)/N the critical fraction qc at the peak of the relative size of second largest component. (PDF) [file pone.0327203.s019.pdf]

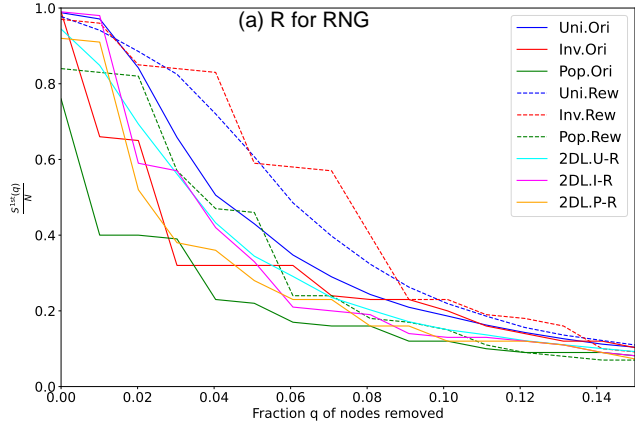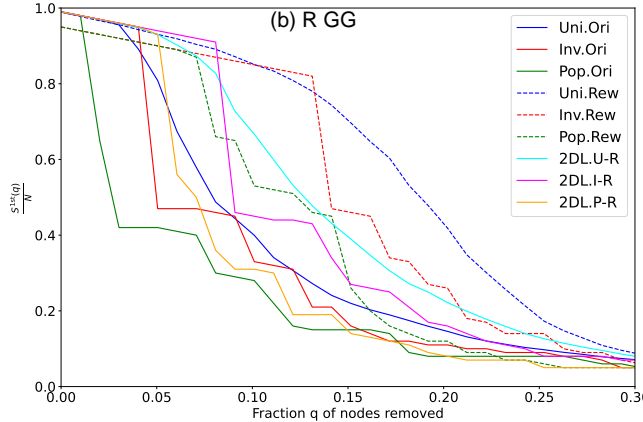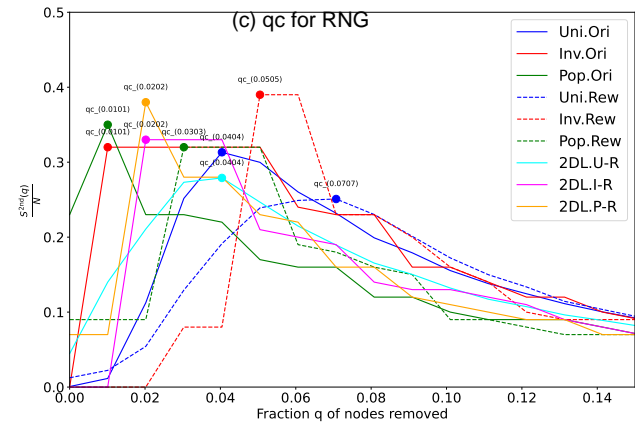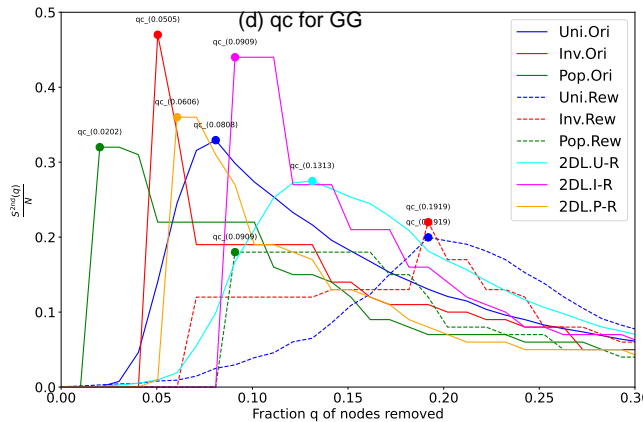

Supplement: S20 Fig — Robustness against recalculated betweenness (RB) attacks in Tokyo networks with N = 100 nodes. For both Rew (Randomized networks) and 2DL lines, the rewiring process preserves the original degree distributions. Two measures are applied: (a) (b) S1st(q)/N the relative size of largest connected component, and (c) (d) S2nd(q)/N the critical fraction qc at the peak of the relative size of second largest component. (PDF) [file pone.0327203.s020.pdf]

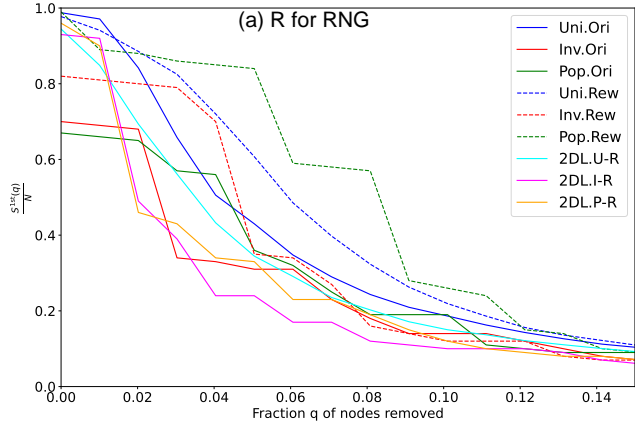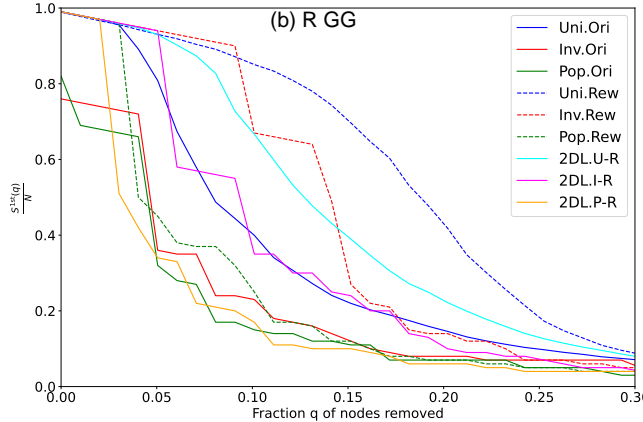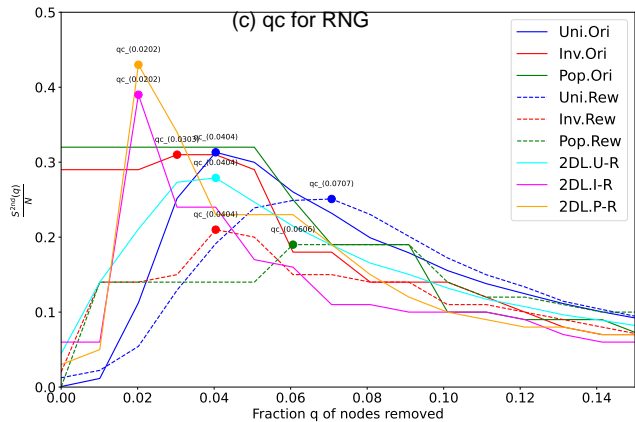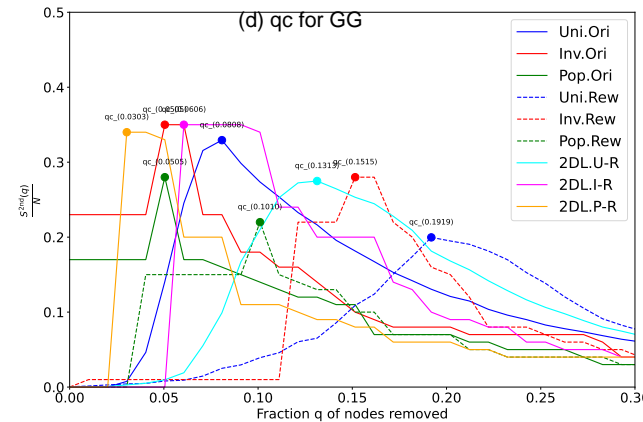

Supplement: S21 Fig — Robustness against recalculated betweenness (RB) attacks for Sendai networks with N = 100 nodes. For both Rew (Randomized networks) and 2DL lines, the rewiring process preserves the original degree distributions. Two measures are applied: (a) (b) S1st(q)/N the relative size of largest connected component, and (c) (d) S2nd(q)/N the critical fraction qc at the peak of the relative size of second largest component. (PDF) [file pone.0327203.s021.pdf]

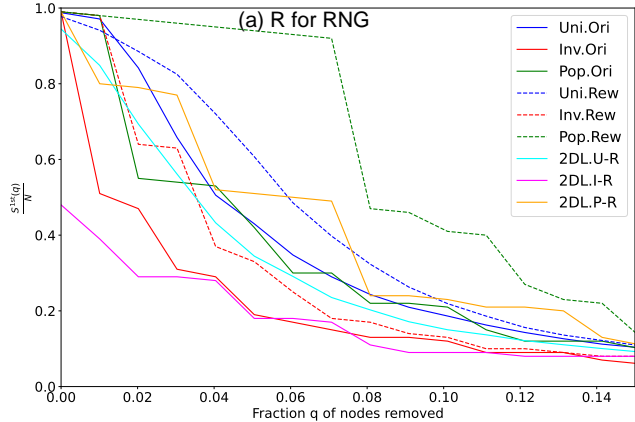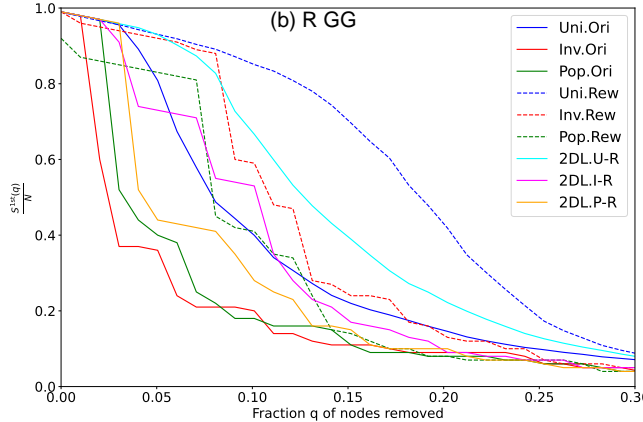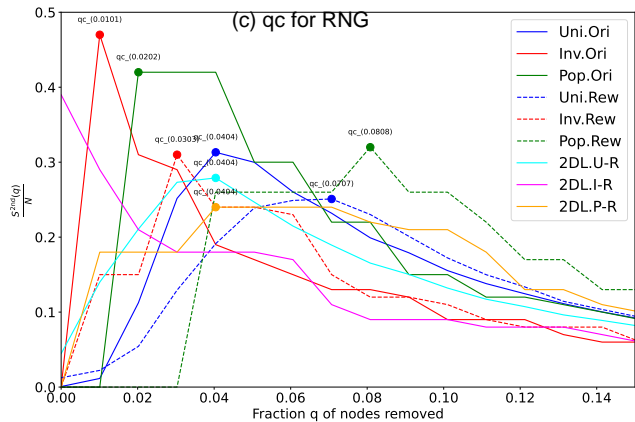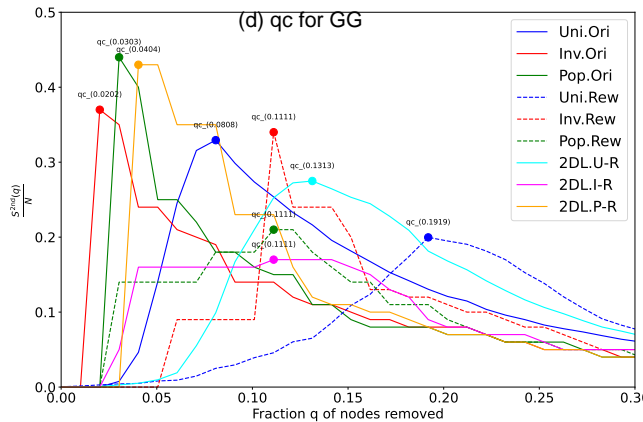

Supplement: S22 Fig — Robustness against recalculated betweenness (RB) attacks for Sapporo networks with N = 100 nodes. For both Rew (Randomized networks) and 2DL lines, the rewiring process preserves the original degree distributions. Two measures are applied: (a) (b) S1st(q)/N the relative size of largest connected component, and (c) (d) S2nd(q)/N the critical fraction qc at the peak of the relative size of second largest component. (PDF) [file pone.0327203.s022.pdf]

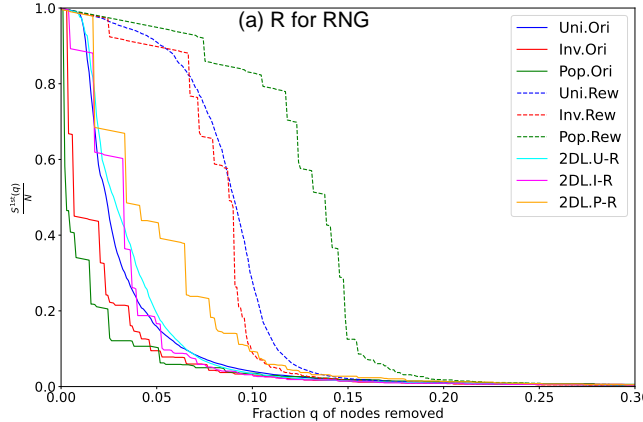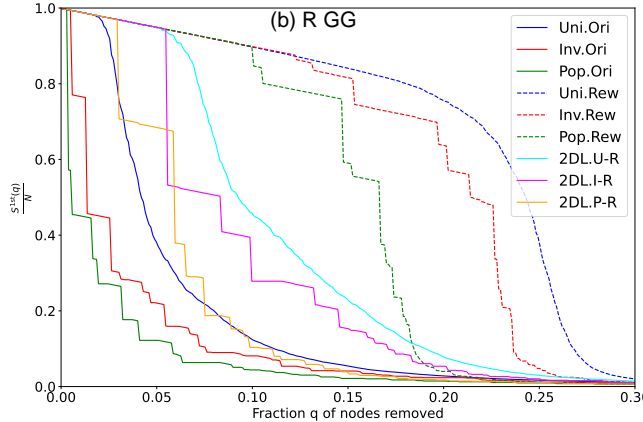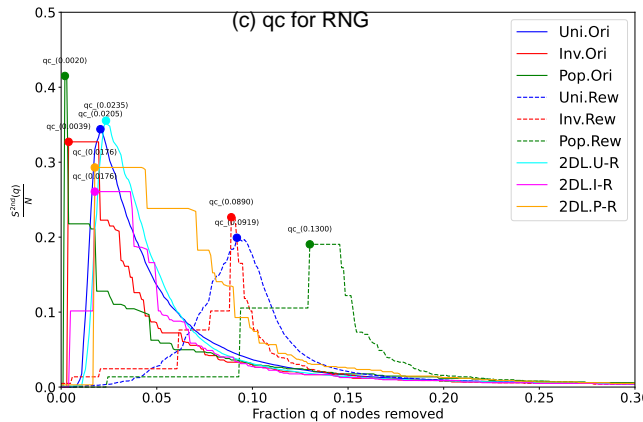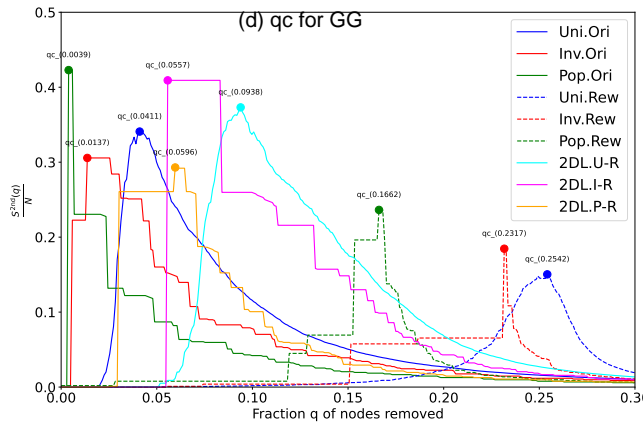

Supplement: S23 Fig — Robustness against recalculated betweenness (RB) attacks for Fukuoka networks with N = 1024 nodes. For both Rew (Randomized networks) and 2DL lines, the rewiring process preserves the original degree distributions. Two measures are applied: (a) (b) S1st(q)/N the relative size of largest connected component, and (c) (d) S2nd(q)/N the critical fraction qc at the peak of the relative size of second largest component. (PDF) [file pone.0327203.s023.pdf]

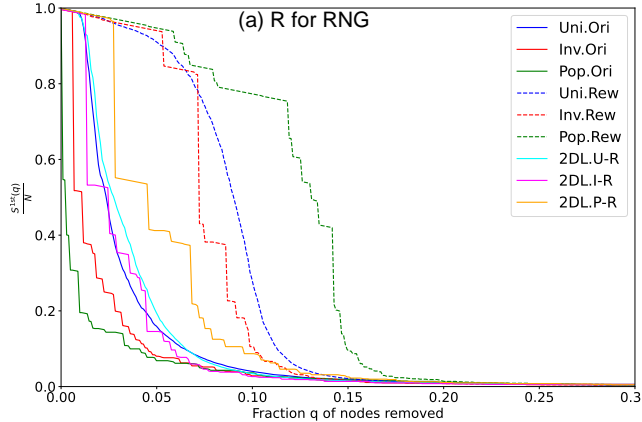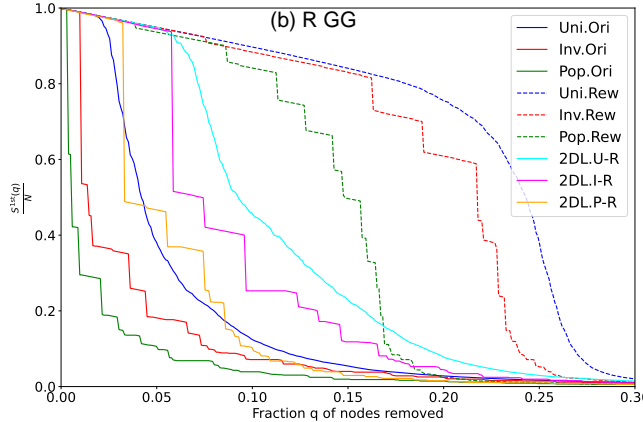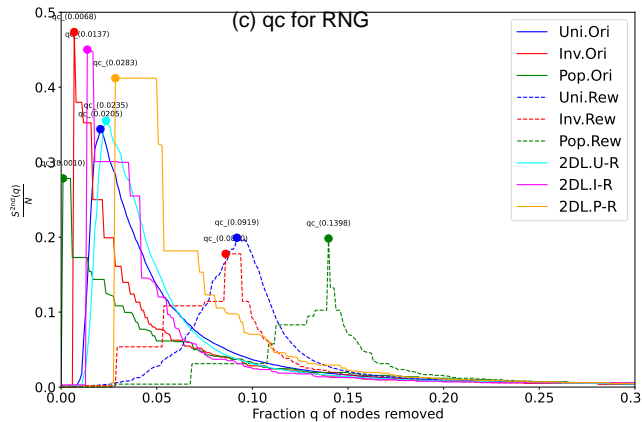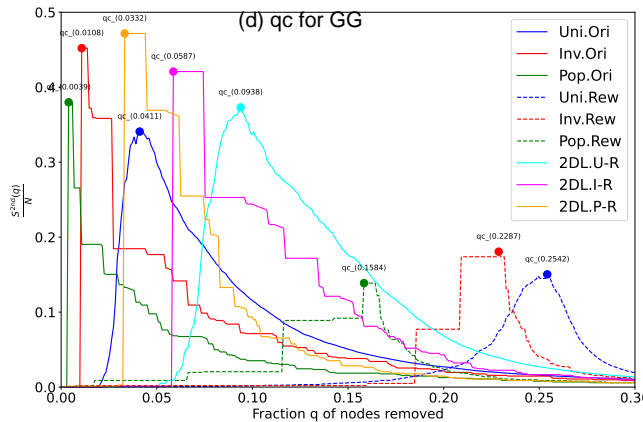

Supplement: S24 Fig — Robustness against recalculated betweenness (RB) attacks for Hiroshima networks with N = 1024 nodes. For both Rew (Randomized networks) and 2DL lines, the rewiring process preserves the original degree distributions. Two measures are applied: (a) (b) S1st(q)/N the relative size of largest connected component, and (c) (d) S2nd(q)/N the critical fraction qc at the peak of the relative size of second largest component. (PDF) [file pone.0327203.s024.pdf]

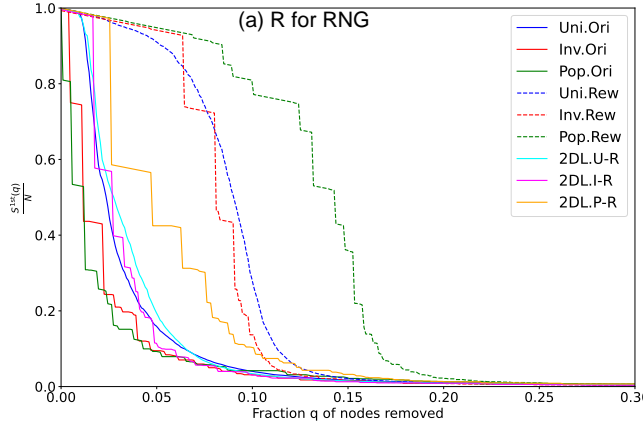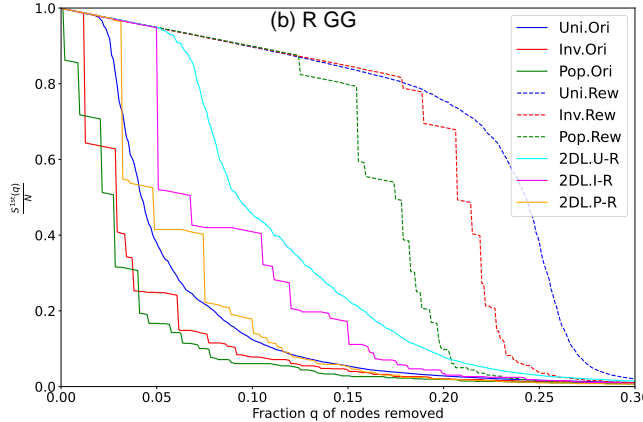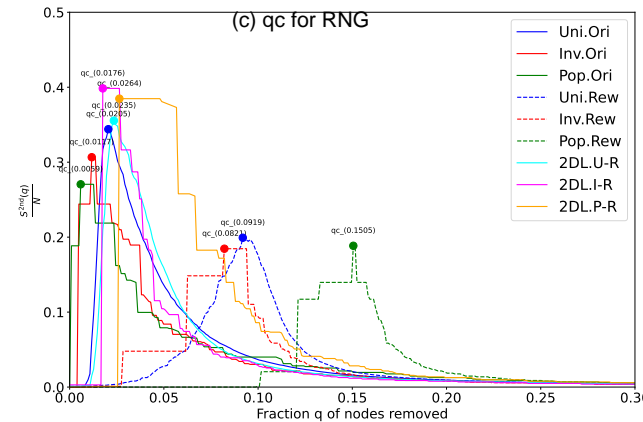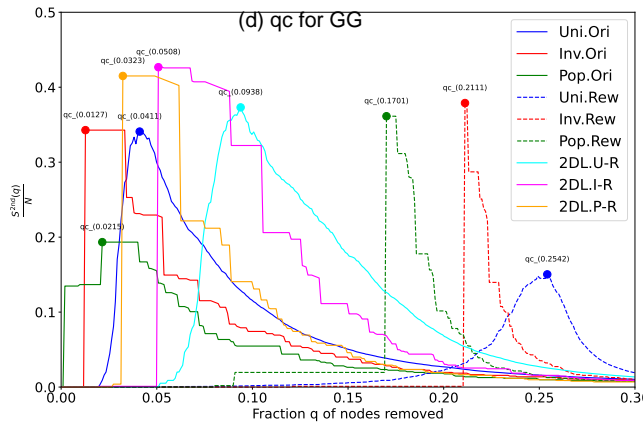

Supplement: S25 Fig — Robustness against recalculated betweenness (RB) attacks for Keihan networks with N = 1024 nodes. For both Rew (Randomized networks) and 2DL lines, the rewiring process preserves the original degree distributions. Two measures are applied: (a) (b) S1st(q)/N the relative size of largest connected component, and (c) (d) S2nd(q)/N the critical fraction qc at the peak of the relative size of second largest component. (PDF) [file pone.0327203.s025.pdf]

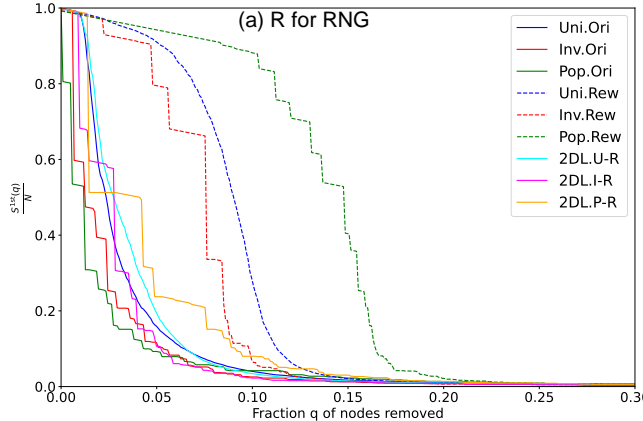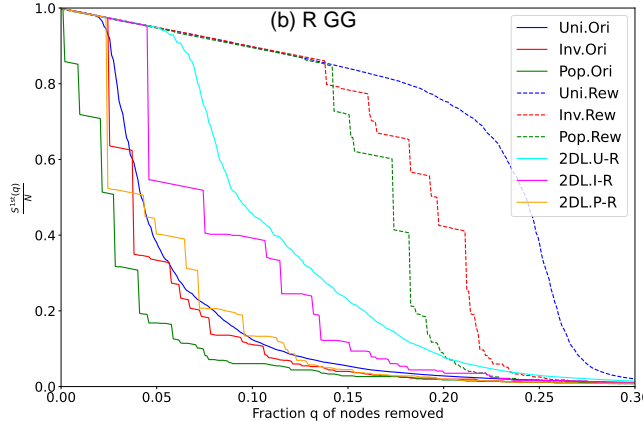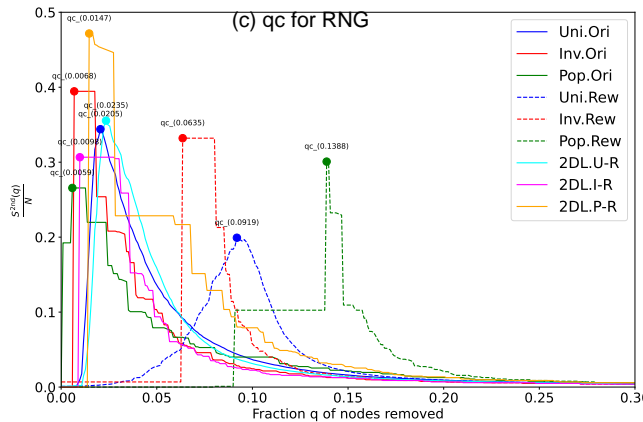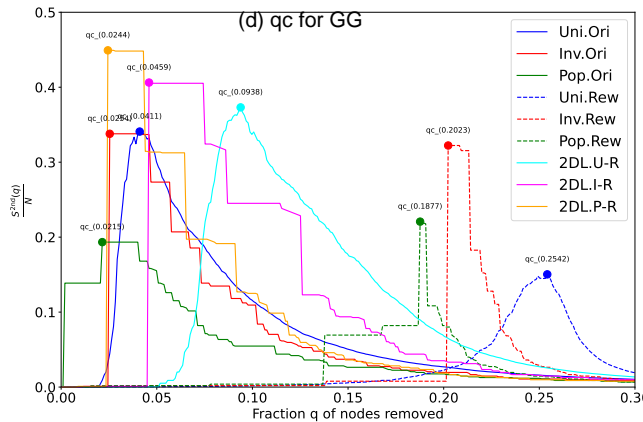

Supplement: S26 Fig — Robustness against recalculated betweenness (RB) attacks for Nagoya networks with N = 1024 nodes. For both Rew (Randomized networks) and 2DL lines, the rewiring process preserves the original degree distributions. Two measures are applied: (a) (b) S1st(q)/N the relative size of largest connected component, and (c) (d) S2nd(q)/N the critical fraction qc at the peak of the relative size of second largest component. (PDF) [file pone.0327203.s026.pdf]

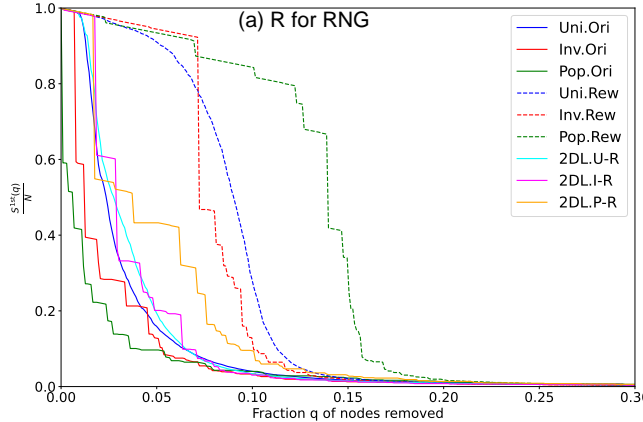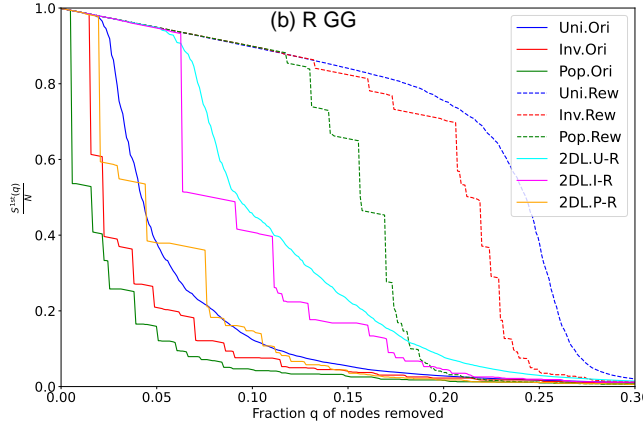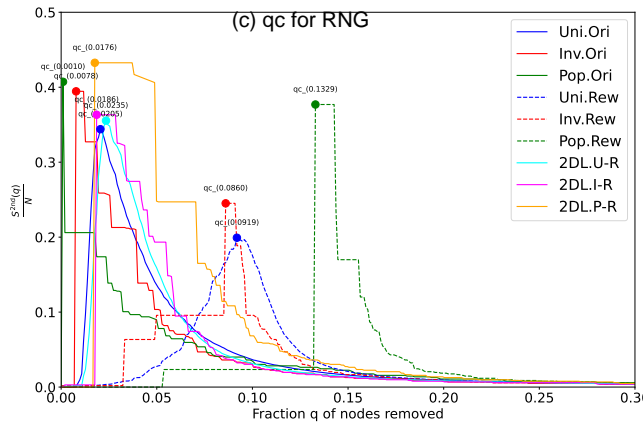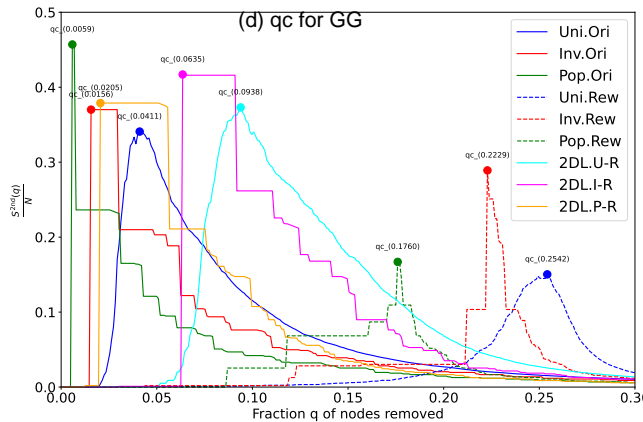

Supplement: S27 Fig — Robustness against recalculated betweenness (RB) attacks for Sendai networks with N = 1024 nodes. For both Rew (Randomized networks) and 2DL lines, the rewiring process preserves the original degree distributions. Two measures are applied: (a) (b) S1st(q)/N the relative size of largest connected component, and (c) (d) S2nd(q)/N the critical fraction qc at the peak of the relative size of second largest component. (PDF) [file pone.0327203.s027.pdf]

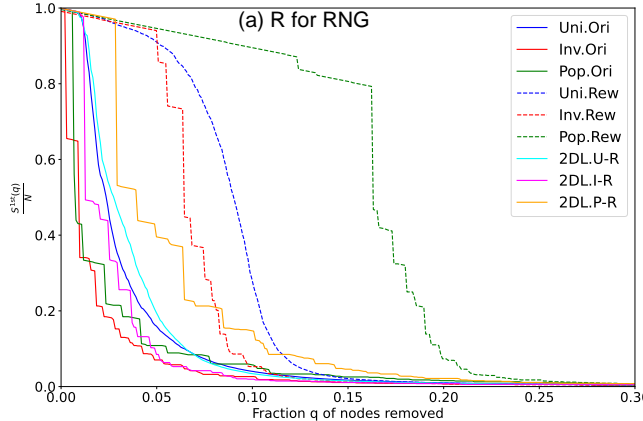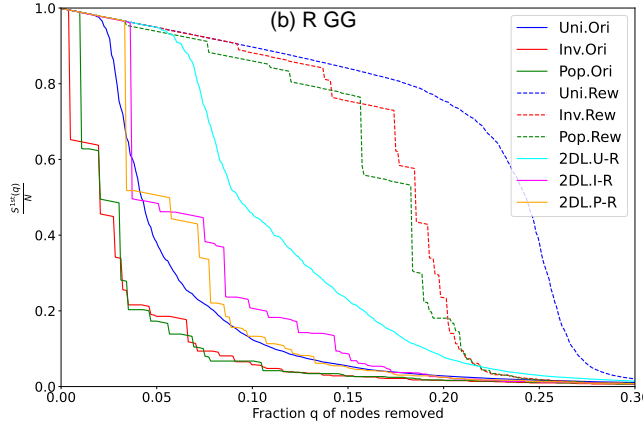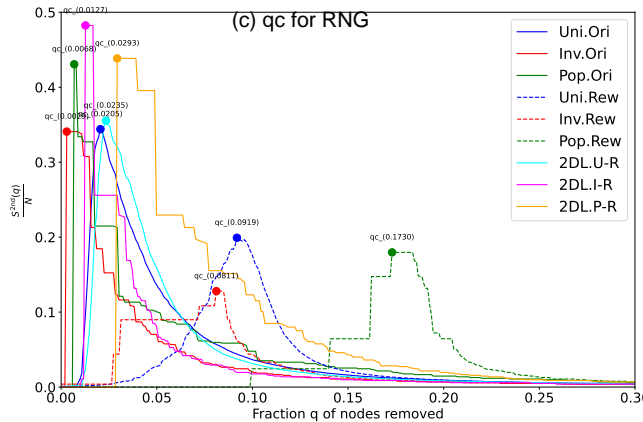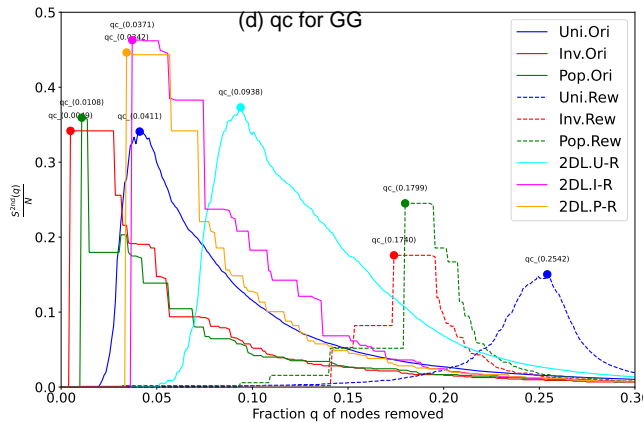

Supplement: S28 Fig — Robustness against recalculated betweenness (RB) attacks for Sapporo networks with N = 1024 nodes. For both Rew (Randomized networks) and 2DL lines, the rewiring process preserves the original degree distributions. Two measures are applied: (a) (b) S1st(q)/N the relative size of largest connected component, and (c) (d) S2nd(q)/N the critical fraction qc at the peak of the relative size of second largest component. (PDF) [file pone.0327203.s028.pdf]

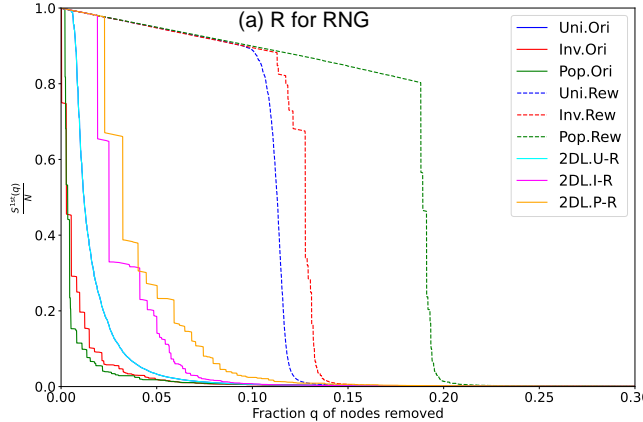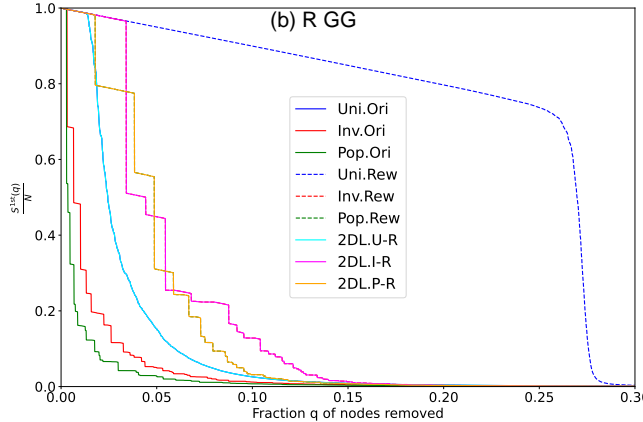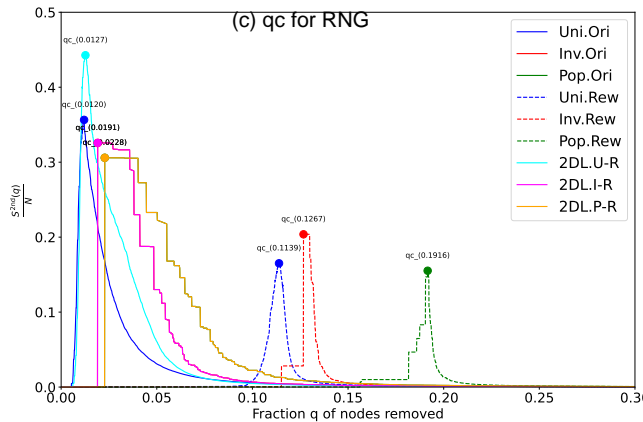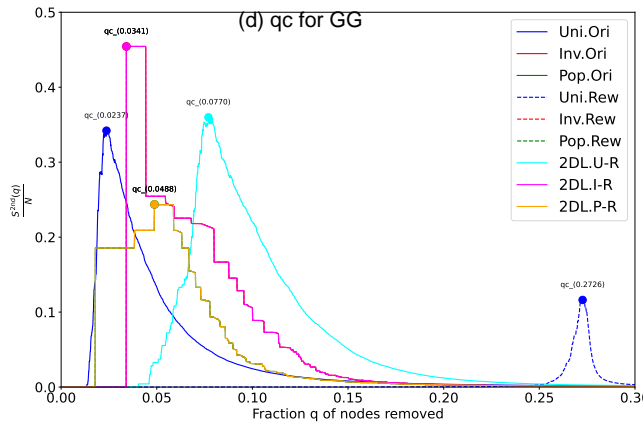

Supplement: S29 Fig — Robustness against recalculated betweenness (RB) attacks for Fukuoka networks with N = 10000 nodes. For both Rew (Randomized networks) and 2DL lines, the rewiring process preserves the original degree distributions. Two measures are applied: (a) (b) S1st(q)/N the relative size of largest connected component, and (c) (d) S2nd(q)/N the critical fraction qc at the peak of the relative size of second largest component. (PDF) [file pone.0327203.s029.pdf]

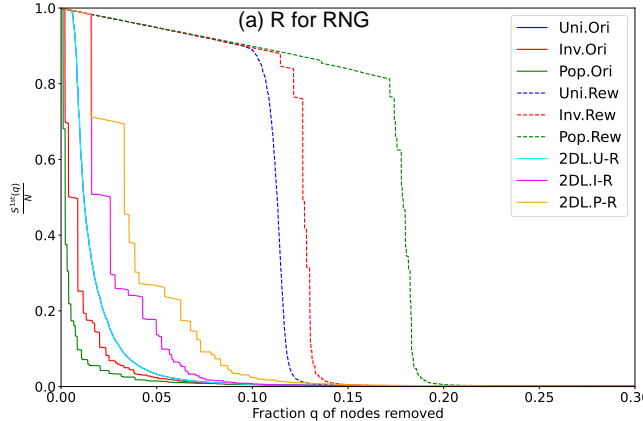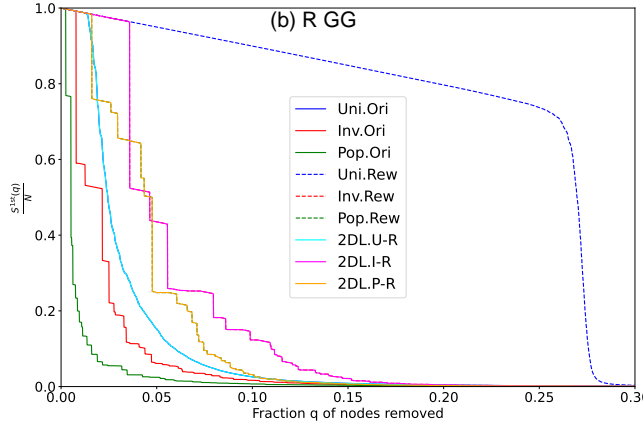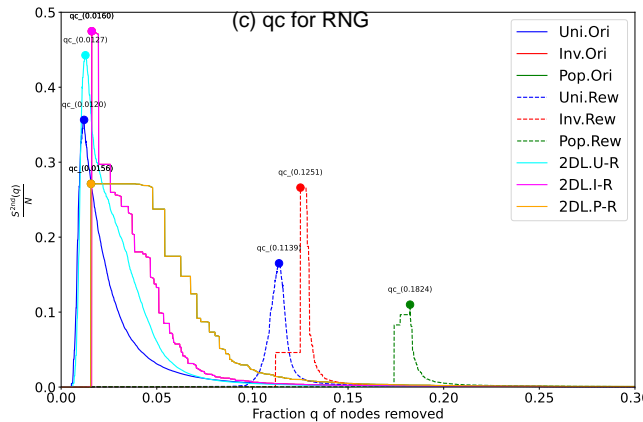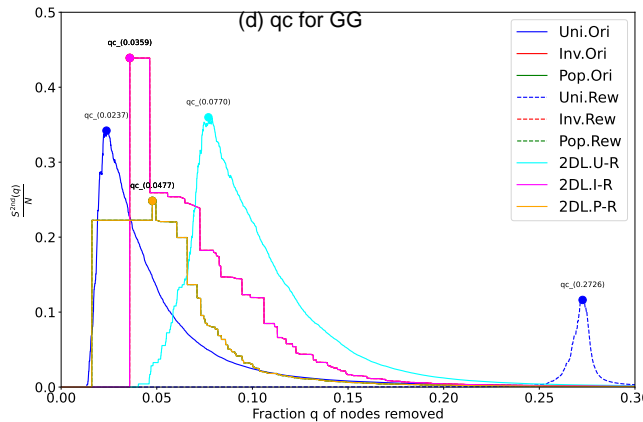

Supplement: S30 Fig — Robustness against recalculated betweenness (RB) attacks for Hiroshima networks with N = 10000 nodes. For both Rew (Randomized networks) and 2DL lines, the rewiring process preserves the original degree distributions. Two measures are applied: (a) (b) S1st(q)/N the relative size of largest connected component, and (c) (d) S2nd(q)/N the critical fraction qc at the peak of the relative size of second largest component. (PDF) [file pone.0327203.s030.pdf]

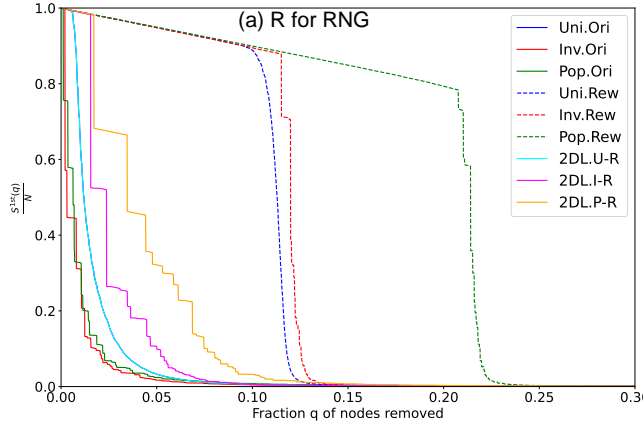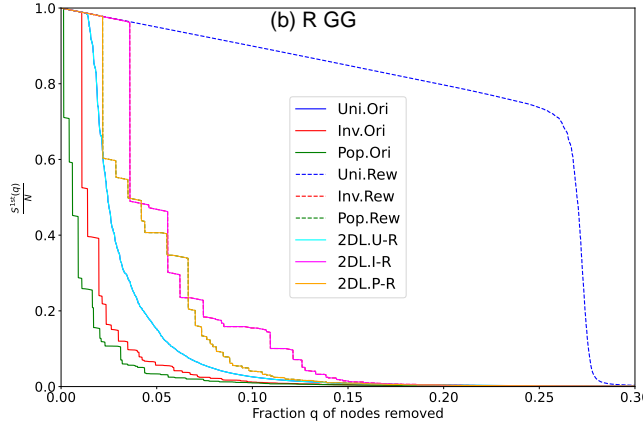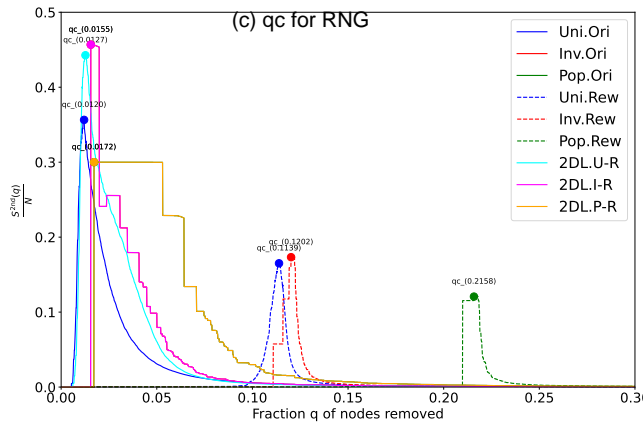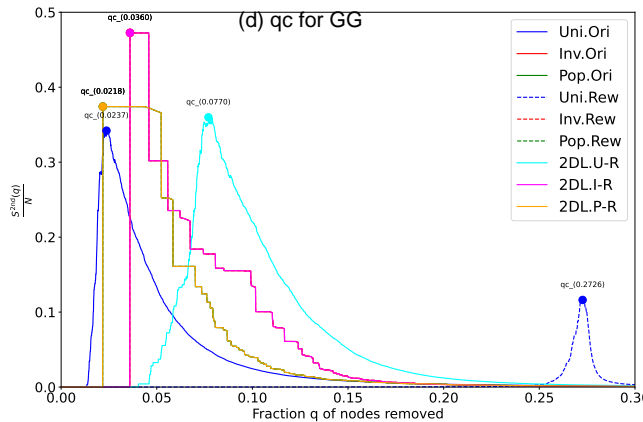

Supplement: S31 Fig — Robustness against recalculated betweenness (RB) attacks for Keihan networks with N = 10000 nodes. For both Rew (Randomized networks) and 2DL lines, the rewiring process preserves the original degree distributions. Two measures are applied: (a) (b) S1st(q)/N the relative size of largest connected component, and (c) (d) S2nd(q)/N the critical fraction qc at the peak of the relative size of second largest component. (PDF) [file pone.0327203.s031.pdf]

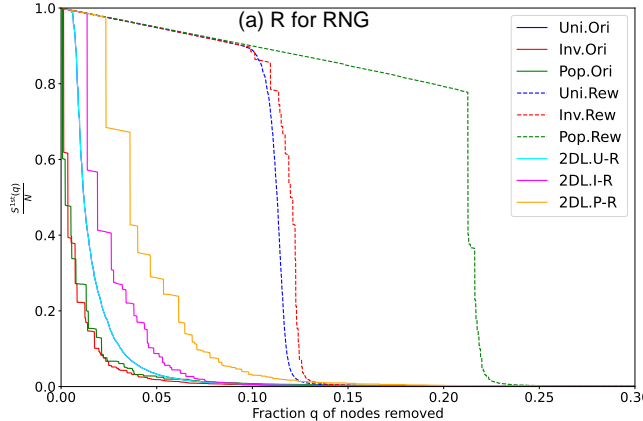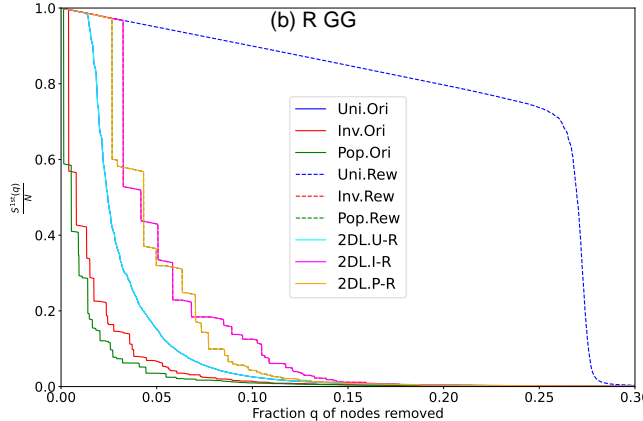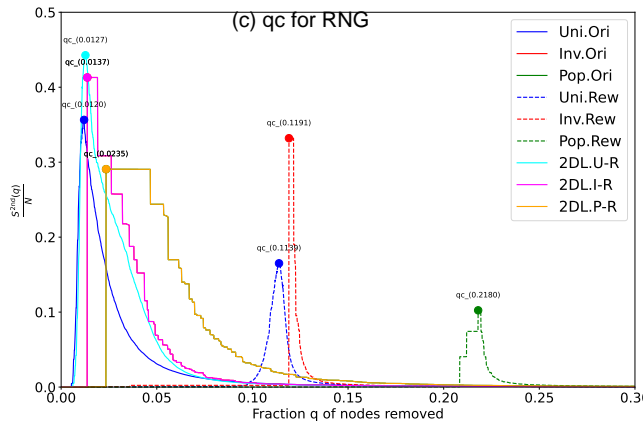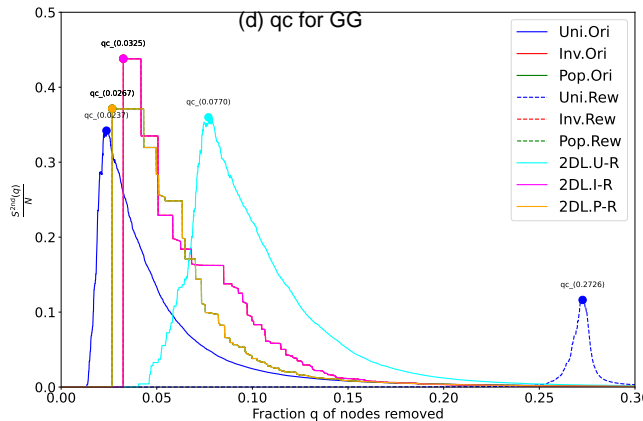

Supplement: S32 Fig — Robustness against recalculated betweenness (RB) attacks for Nagoya networks with N = 10000 nodes. For both Rew (Randomized networks) and 2DL lines, the rewiring process preserves the original degree distributions. Two measures are applied: (a) (b) S1st(q)/N the relative size of largest connected component, and (c) (d) S2nd(q)/N the critical fraction qc at the peak of the relative size of second largest component. (PDF) [file pone.0327203.s032.pdf]

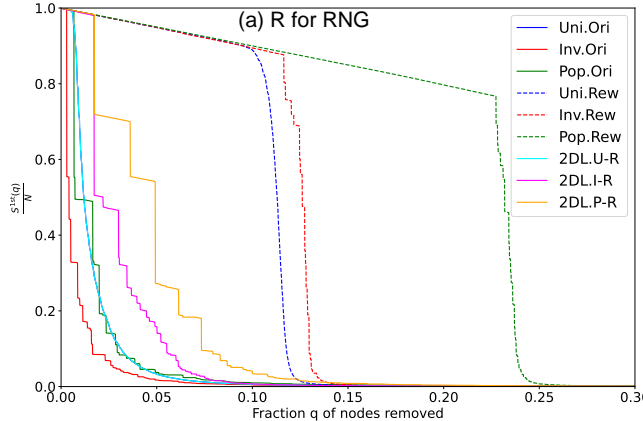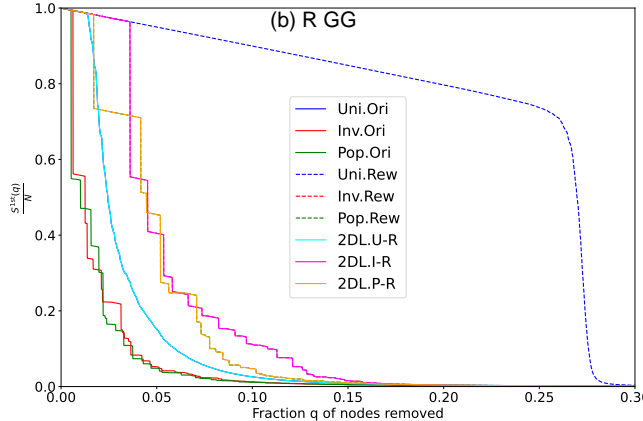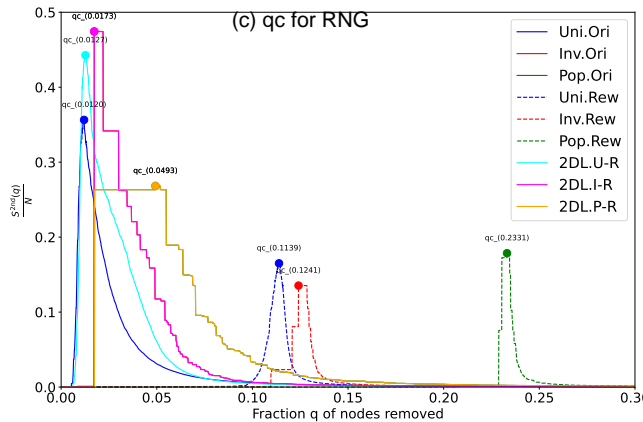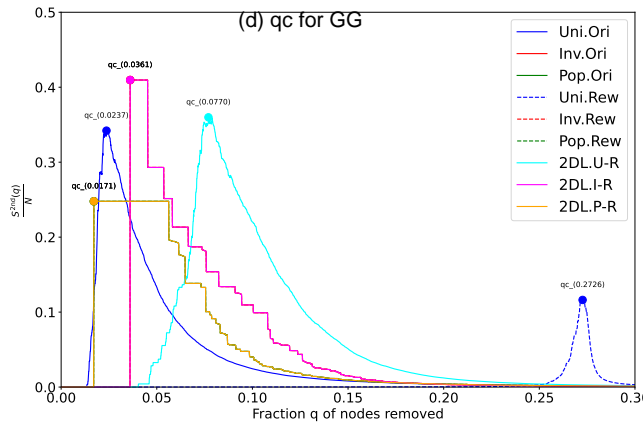

Supplement: S33 Fig — Robustness against recalculated betweenness (RB) attacks in Tokyo networks with N = 10000 nodes. For both Rew (Randomized networks) and 2DL lines, the rewiring process preserves the original degree distributions. Two measures are applied: (a) (b) S1st(q)/N the relative size of largest connected component, and (c) (d) S2nd(q)/N the critical fraction qc at the peak of the relative size of second largest component. (PDF) [file pone.0327203.s033.pdf]

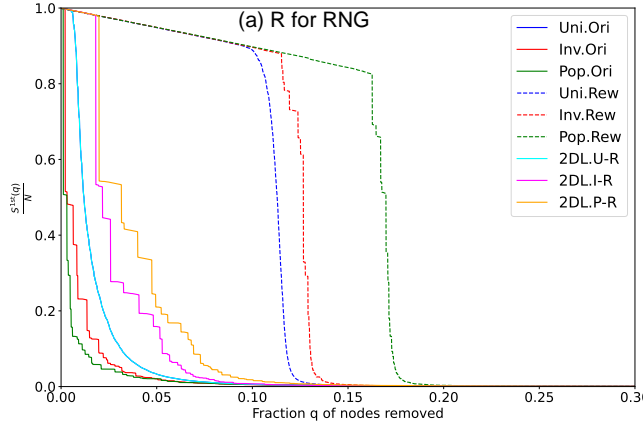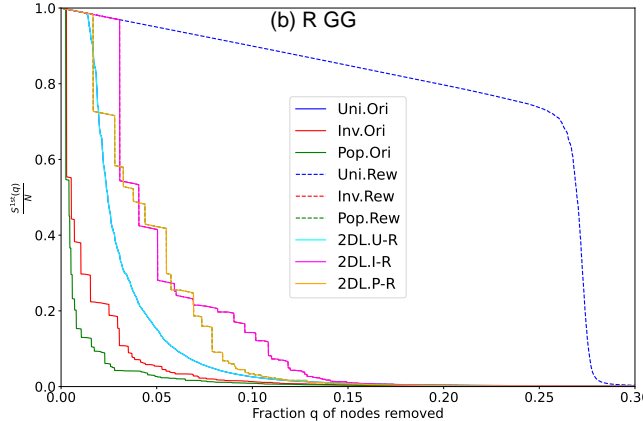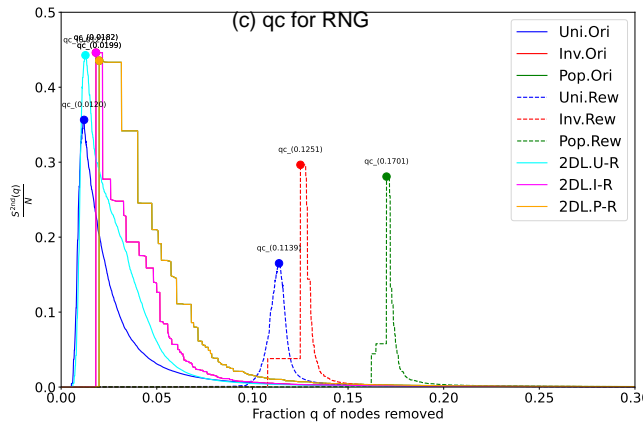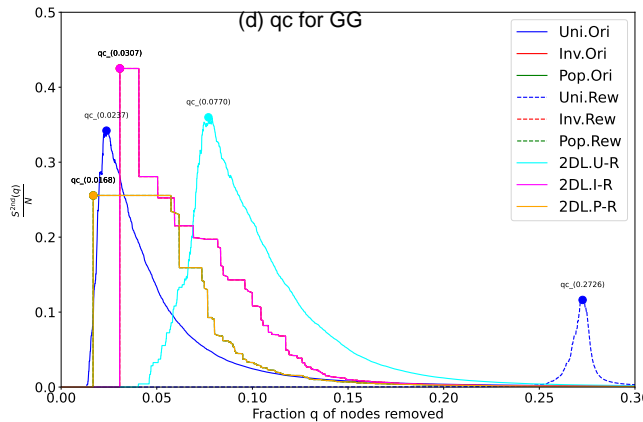

Supplement: S34 Fig — Robustness against recalculated betweenness (RB) attacks for Sendai networks with N = 10000 nodes. For both Rew (Randomized networks) and 2DL lines, the rewiring process preserves the original degree distributions. Two measures are applied: (a) (b) S1st(q)/N the relative size of largest connected component, and (c) (d) S2nd(q)/N the critical fraction qc at the peak of the relative size of second largest component. (PDF) [file pone.0327203.s034.pdf]

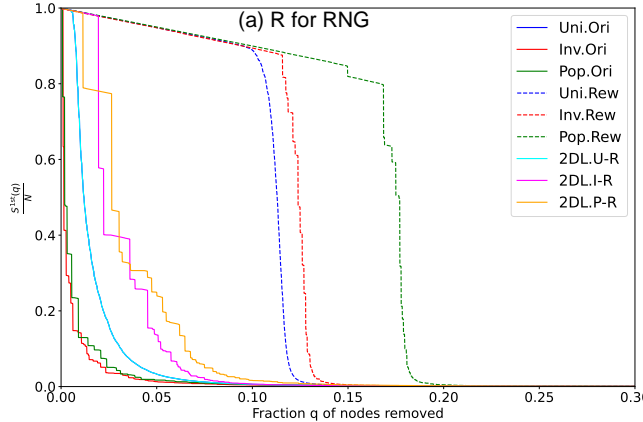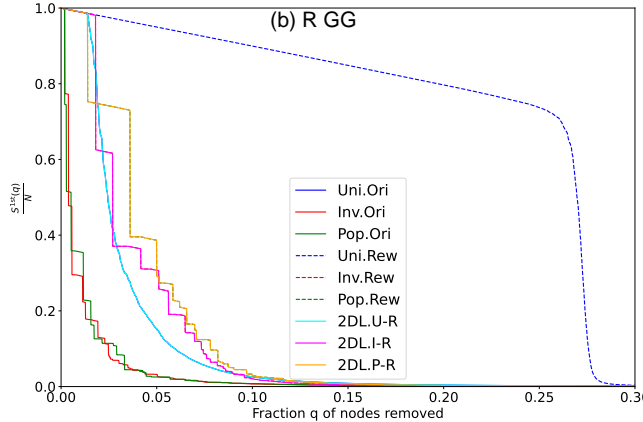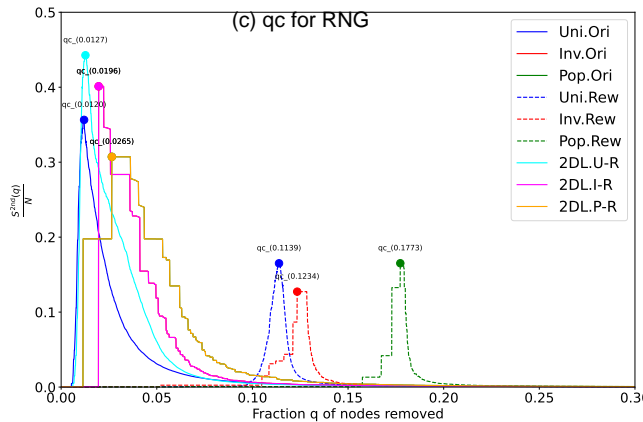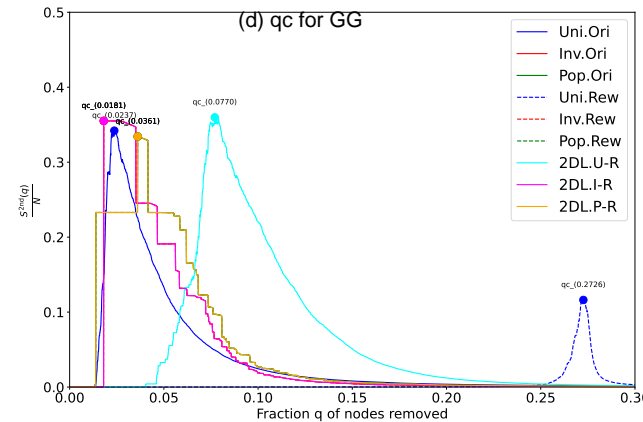

Supplement: S35 Fig — Robustness against recalculated betweenness (RB) attacks for Sapporo networks with N = 10000 nodes. For both Rew (Randomized networks) and 2DL lines, the rewiring process preserves the original degree distributions. Two measures are applied: (a) (b) S1st(q)/N the relative size of largest connected component, and (c) (d) S2nd(q)/N the critical fraction qc at the peak of the relative size of second largest component. (PDF) [file pone.0327203.s035.pdf]

(a) RNG

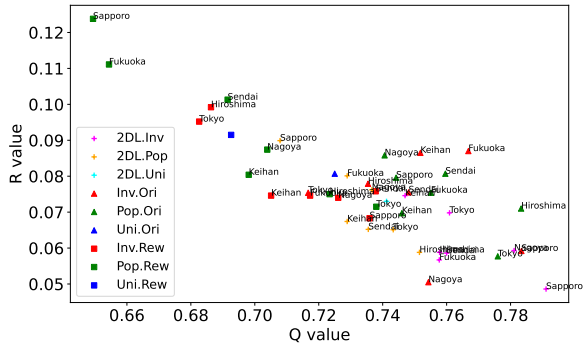

(b) GG

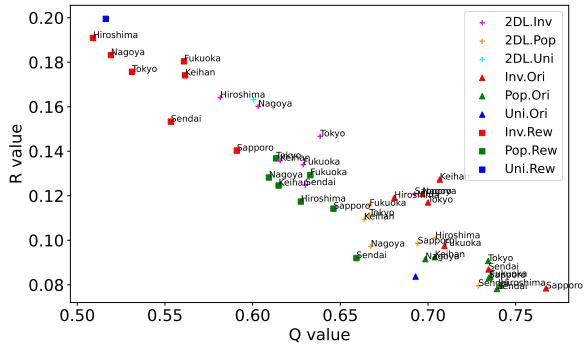

Supplement: S36 Fig — Relation between robustness index RRB and modularity Q in networks with N = 100 nodes. (PDF) [file pone.0327203.s036.pdf]

(a) RNG

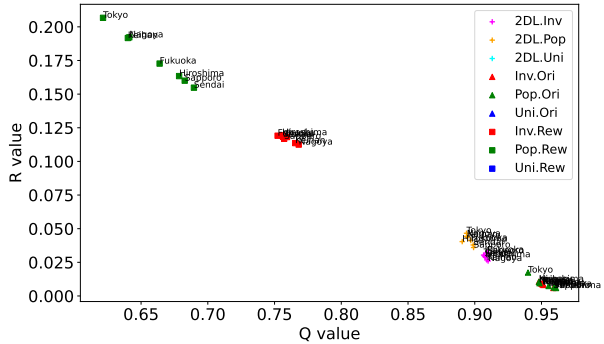

(b) GG

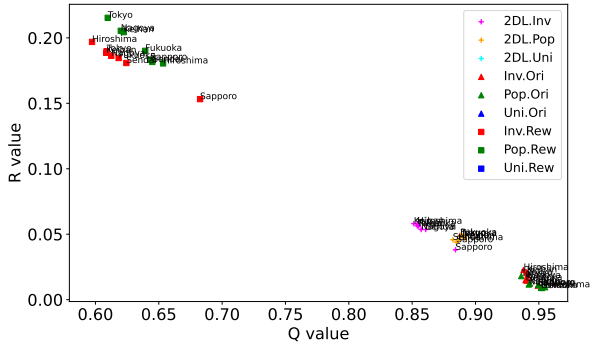

Supplement: S37 Fig — Relation between robustness index RRB and modularity Q in networks with N = 10000 nodes (PDF) [file pone.0327203.s037.pdf]

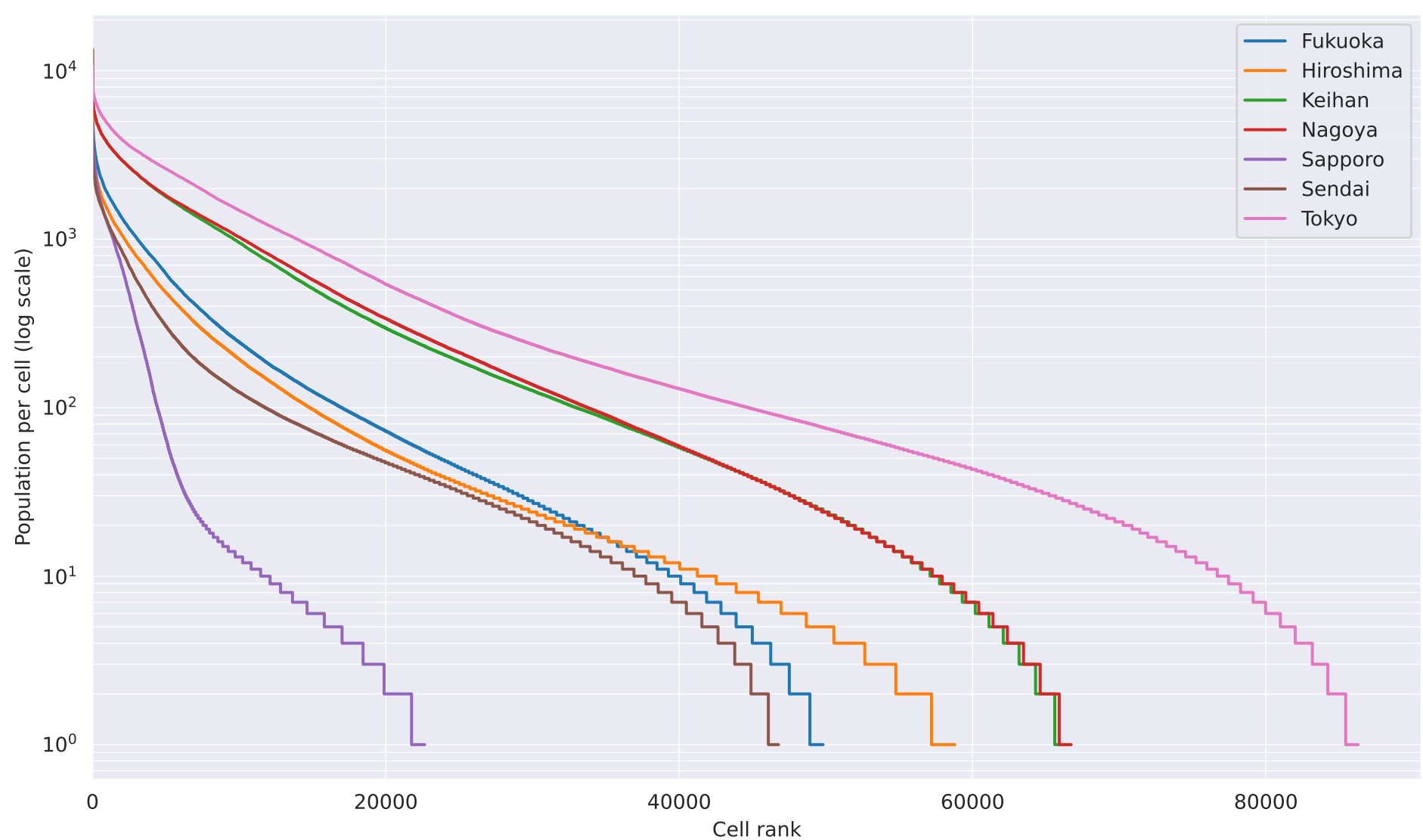

Supplement: S38 Fig — Semi-logarithmic plot of population per 500m×500m block meshes in seven major Japanese areas, with meshes sorted in decreasing order of population. Each curve represents an area. The linear decay on the logarithmic scale indicates that a small number of meshes concentrate the majority of the urban population. This heavy-tailed distribution supports the use of rank-based node selections for the Pop. and Inv. networks. (PDF) [file pone.0327203.s038.pdf]
