# Supplementary material for: Vulnerable connectivity caused by local communities in spatial networks
Source: PLoS One. 2025 Jul 2;20(7):e0327203. doi: 10.1371/journal.pone.0327203 (PMC12221043; doi:10.1371/journal.pone.0327203)
Supplement: S1 Table — Robustness index (RRB) and Critical fraction (qcRB) against Recalculated Betweenness (RB) attacks in networks with N = 100 nodes for seven major Japanese areas. Higher values indicate greater robustness of connectivity. For RRB, values with upper-triangles (△) indicate higher robustness than Uni. case, while values with lower-triangles (▿) indicate lower robustness than Uni. case. For qcRB, all values are marked with lower-triangles (▿) as they show lower robustness than Uni. case. (PDF) [file pone.0327203.s039.pdf]

Vulnerable connectivity caused by local communities in spatial networks

Yingzhou MOU[1*] and Yukio HAYASHI[1]

[1]Japan Advanced Institute of Science and Technology, Nomi-city, Ishikawa 923-1292, Japan

* mouyingzhou@outlook.com

Abstract

Local communities by concentration of nodes connected with short links are widely observed in spatial networks. However, how such structure affects robustness of connectivity against malicious attacks remains unclear. This study investigates the impact of local communities on the robustness by modeling planar infrastructure networks whose node's locations are based on statistical population data. Our research reveals that the robustness is weakened by strong local communities in spatial networks. These results highlight the potential of long-distance links in mitigating the negative effects of local community on the robustness.

Table S1

Cities	R^{RB}				q_c^{RB}			
	RNG		GG		RNG		GG	
	Inv.	Pop.	Inv.	Pop.	Inv.	Pop.	Inv.	Pop.
Fukuoka	$0.0870^{\triangle}$	$0.0754^{\triangledown}$	$0.0975^{\triangle}$	0.0837	$0.0101^{\triangledown}$	$0.0202^{\triangledown}$	$0.0303^{\triangledown}$	$0.0404^{\triangledown}$
Hiroshima	$0.0779^{\triangledown}$	$0.0710^{\triangledown}$	$0.1191^{\triangle}$	$0.0796^{\triangledown}$	$0.0101^{\triangledown}$	$0.0101^{\triangledown}$	$0.0404^{\triangledown}$	$0.0202^{\triangledown}$
Keihan	$0.0865^{\triangle}$	$0.0697^{\triangledown}$	$0.1273^{\triangle}$	$0.0924^{\triangle}$	$0.0303^{\triangledown}$	$0.0202^{\triangledown}$	$0.0505^{\triangledown}$	$0.0505^{\triangledown}$
Nagoya	$0.0505^{\triangledown}$	$0.0858^{\triangle}$	$0.1208^{\triangle}$	$0.0915^{\triangle}$	$0.0202^{\triangledown}$	$0.0202^{\triangledown}$	$0.0505^{\triangledown}$	$0.0505^{\triangledown}$
Tokyo	$0.0754^{\triangledown}$	$0.0577^{\triangledown}$	$0.1170^{\triangle}$	$0.0907^{\triangle}$	$0.0101^{\triangledown}$	$0.0101^{\triangledown}$	$0.0505^{\triangledown}$	$0.0202^{\triangledown}$
Sendai	$0.0755^{\triangledown}$	0.0807	$0.0869^{\triangle}$	$0.0781^{\triangledown}$	$0.0303^{\triangledown}$	$0.0000^{\triangledown}$	$0.0505^{\triangledown}$	$0.0505^{\triangledown}$
Sapporo	$0.0592^{\triangledown}$	$0.0796^{\triangledown}$	$0.0784^{\triangledown}$	$0.0832^{\triangledown}$	$0.0101^{\triangledown}$	$0.0202^{\triangledown}$	$0.0202^{\triangledown}$	$0.0303^{\triangledown}$
Uniform	0.0807		0.0837		0.0404		0.0808	
2D Lattice	0.1636				0.0909			
