# Supplementary material for: Vulnerable connectivity caused by local communities in spatial networks
Source: PLoS One. 2025 Jul 2;20(7):e0327203. doi: 10.1371/journal.pone.0327203 (PMC12221043; doi:10.1371/journal.pone.0327203)
Supplement: S3 Table — Robustness index (RRB) and Critical fraction (qcRB) against Recalculated Betweenness (RB) attacks in networks with N = 10000 nodes for seven major Japanese areas. Higher values indicate greater robustness of connectivity. Values with lower-triangles (▿) indicate where the cases of Pop. and Inv. have lower robustness of connectivity than the cases of Uni. for both RNG and GG. (PDF) [file pone.0327203.s041.pdf]

Vulnerable connectivity caused by local communities in spatial networks

Yingzhou MOU[1*] and Yukio HAYASHI[1]

[1]Japan Advanced Institute of Science and Technology, Nomi-city, Ishikawa 923-1292, Japan

* mouyingzhou@outlook.com

Abstract

Local communities by concentration of nodes connected with short links are widely observed in spatial networks. However, how such structure affects robustness of connectivity against malicious attacks remains unclear. This study investigates the impact of local communities on the robustness by modeling planar infrastructure networks whose node's locations are based on statistical population data. Our research reveals that the robustness is weakened by strong local communities in spatial networks. These results highlight the potential of long-distance links in mitigating the negative effects of local community on the robustness.

Table S3

Cities	R^{RB}				q_c^{RB}			
	RNG		GG		RNG		GG	
	Inv.	Pop.	Inv.	Pop.	Inv.	Pop.	Inv.	Pop.
Fukuoka	0.0082$^{\triangledown}$	0.0070$^{\triangledown}$	0.0149$^{\triangledown}$	0.0091$^{\triangledown}$	0.0028$^{\triangledown}$	0.0045$^{\triangledown}$	0.0032$^{\triangledown}$	0.0029$^{\triangledown}$
Hiroshima	0.0106$^{\triangledown}$	0.0059$^{\triangledown}$	0.0227$^{\triangledown}$	0.0095$^{\triangledown}$	0.0021$^{\triangledown}$	0.0011$^{\triangledown}$	0.0079$^{\triangledown}$	0.0052$^{\triangledown}$
Keihan	0.0090$^{\triangledown}$	0.0105$^{\triangledown}$	0.0208$^{\triangledown}$	0.0123$^{\triangledown}$	0.0021$^{\triangledown}$	0.0012$^{\triangledown}$	0.0109$^{\triangledown}$	0.0014$^{\triangledown}$
Nagoya	0.0086$^{\triangledown}$	0.0095$^{\triangledown}$	0.0166$^{\triangledown}$	0.0116$^{\triangledown}$	0.0014$^{\triangledown}$	0.0008$^{\triangledown}$	0.0040$^{\triangledown}$	0.0014$^{\triangledown}$
Tokyo	0.0092$^{\triangledown}$	0.0174$^{\triangledown}$	0.0189$^{\triangledown}$	0.0180$^{\triangledown}$	0.0030$^{\triangledown}$	0.0068$^{\triangledown}$	0.0063$^{\triangledown}$	0.0053$^{\triangledown}$
Sendai	0.0094$^{\triangledown}$	0.0063$^{\triangledown}$	0.0146$^{\triangledown}$	0.0089$^{\triangledown}$	0.0022$^{\triangledown}$	0.0012$^{\triangledown}$	0.0029$^{\triangledown}$	0.0025$^{\triangledown}$
Sapporo	0.0059$^{\triangledown}$	0.0074$^{\triangledown}$	0.0108$^{\triangledown}$	0.0112$^{\triangledown}$	0.0011$^{\triangledown}$	0.0019$^{\triangledown}$	0.0039$^{\triangledown}$	0.0019$^{\triangledown}$
Uniform	0.0173		0.0334		0.0115		0.0229	
2D Lattice	0.0647				0.0679			
