# Supplementary material for: Vulnerable connectivity caused by local communities in spatial networks
Source: PLoS One. 2025 Jul 2;20(7):e0327203. doi: 10.1371/journal.pone.0327203 (PMC12221043; doi:10.1371/journal.pone.0327203)
Supplement: S9 Table — One-way ANOVA results evaluating the influence of node distribution (Population-based, Inverse population-based, and Uniform) on the robustness index (R) and the critical fraction (qc) against Initial Degree (ID) attacks (N = 1024 nodes). F-value indicates the ratio of between-group variance to within-group variance. p-value shows the probability that the observed group differences are due to chance (typically, p<0.05 is considered statistically significant). η2 denotes the effect size, indicating the proportion of variance explained by the group factor. In this table, the qc result for RNG networks shows no significant difference (p = 0.249). This may be due to two factors: (1) the higher average degree ⟨k⟩ in RNG Pop. networks (see Table 4), which offers more alternative paths after node removal; and (2) the presence of grid-like substructures in Pop. networks, which can enhance local community and delay critical fragmentation. (PDF) [file pone.0327203.s047.pdf]

Vulnerable connectivity caused by local communities in spatial networks

Yingzhou MOU[1*] and Yukio HAYASHI[1]

[1]Japan Advanced Institute of Science and Technology, Nomi-city, Ishikawa 923-1292, Japan

* mouyingzhou@outlook.com

Abstract

Local communities by concentration of nodes connected with short links are widely observed in spatial networks. However, how such structure affects robustness of connectivity against malicious attacks remains unclear. This study investigates the impact of local communities on the robustness by modeling planar infrastructure networks whose node's locations are based on statistical population data. Our research reveals that the robustness is weakened by strong local communities in spatial networks. These results highlight the potential of long-distance links in mitigating the negative effects of local community on the robustness.

Table S9

**Network**	**Metric**	**F-value**	**p-value**	η^2	**Distributional Differences**
RNG	R^{ID}	13.25	**0.00029**	0.595	Uni. is significantly stronger than Pop. and Inv.
GG	R^{ID}	6.75	**0.0065**	0.429	Uni. > Inv. > Pop.
RNG	${q_c}^{ID}$	1.50	0.249	0.143	*Not significant; inconsistent rankings across cities*
GG	${q_c}^{ID}$	15.76	**0.00011**	0.637	Uni. is significantly stronger than Pop. and Inv.
