# Supplementary material for: Vulnerable connectivity caused by local communities in spatial networks
Source: PLoS One. 2025 Jul 2;20(7):e0327203. doi: 10.1371/journal.pone.0327203 (PMC12221043; doi:10.1371/journal.pone.0327203)
Supplement: S10 Table — One-way ANOVA results evaluating the influence of node distribution (Population-based, Inverse population-based, and Uniform) on the robustness index (R) and the critical fraction (qc) against Random Failures (RF) (N = 1024 nodes). F-value indicates the ratio of between-group variance to within-group variance. p-value shows the probability that the observed group differences are due to chance (typically, p<0.05 is considered statistically significant). η2 denotes the effect size, indicating the proportion of variance explained by the group factor; conventionally, η2>0.14 is considered a large effect. Notably, the R result for RNG networks shows no significant difference (p = 0.104), and the qc result is marginally significant (p = 0.028). These results may be explained by two factors: (1) higher average degree ⟨k⟩, which provides alternative paths after node removal (see Table 4); and (2) the presence of grid-like substructures in certain area of the network, which enhance local community and delay critical fragmentation. (PDF) [file pone.0327203.s048.pdf]

Vulnerable connectivity caused by local communities in spatial networks

Yingzhou MOU[1*] and Yukio HAYASHI[1]

[1]Japan Advanced Institute of Science and Technology, Nomi-city, Ishikawa 923-1292, Japan

* mouyingzhou@outlook.com

Abstract

Local communities by concentration of nodes connected with short links are widely observed in spatial networks. However, how such structure affects robustness of connectivity against malicious attacks remains unclear. This study investigates the impact of local communities on the robustness by modeling planar infrastructure networks whose node's locations are based on statistical population data. Our research reveals that the robustness is weakened by strong local communities in spatial networks. These results highlight the potential of long-distance links in mitigating the negative effects of local community on the robustness.

Table S10

**Network**	**Metric**	**F-value**	**p-value**	η^2	**Distributional Differences**
RNG	R^{RF}	2.58	0.104	0.223	*Not significant; Pop. and Uni. each ranked highest in 3 cities*
GG	R^{RF}	22.62	**0.000012**	0.715	Uni. > Inv. > Pop.
RNG	${q_c}^{RF}$	4.39	**0.028**	0.328	Uni. is significantly stronger than Pop. and Inv.
GG	${q_c}^{RF}$	5.87	**0.0109**	0.395	Uni. is significantly stronger than Pop. and Inv.
