# Supplementary material for: Vulnerable connectivity caused by local communities in spatial networks
Source: PLoS One. 2025 Jul 2;20(7):e0327203. doi: 10.1371/journal.pone.0327203 (PMC12221043; doi:10.1371/journal.pone.0327203)
Supplement: S11 Table — Estimated number of communities in original networks and after node relocation on a 2D lattice (relocated). Values with upper-triangles (△) indicate that Pop.-based or Inv.-based has more communities than Uni.-based networks. Values in bold indicate fewer communities than Uni.-based networks in both RNG and GG. (PDF) [file pone.0327203.s049.pdf]

Vulnerable connectivity caused by local communities in spatial networks

Yingzhou MOU[1*] and Yukio HAYASHI[1]

[1]Japan Advanced Institute of Science and Technology, Nomi-city, Ishikawa 923-1292, Japan

* mouyingzhou@outlook.com

Abstract

Local communities by concentration of nodes connected with short links are widely observed in spatial networks. However, how such structure affects robustness of connectivity against malicious attacks remains unclear. This study investigates the impact of local communities on the robustness by modeling planar infrastructure networks whose node's locations are based on statistical population data. Our research reveals that the robustness is weakened by strong local communities in spatial networks. These results highlight the potential of long-distance links in mitigating the negative effects of local community on the robustness.

Table S11

**Cities**	**Original**				**2D Lattice**			
	RNG		GG		RNG		GG	
	Inv.	Pop.	Inv.	Pop.	Inv.	Pop.	Inv.	Pop.
Fukuoka	$25^{\triangle}$	$24^{\triangle}$	$21^{\triangle}$	$23^{\triangle}$	25	**24**	$17^{\triangle}$	$19^{\triangle}$
Hiroshima	22	$27^{\triangle}$	20	$22^{\triangle}$	$28^{\triangle}$	**23**	$17^{\triangle}$	$20^{\triangle}$
Keihan	22	$23^{\triangle}$	**19**	**19**	$27^{\triangle}$	**22**	16	$19^{\triangle}$
Nagoya	$24^{\triangle}$	22	20	20	$28^{\triangle}$	**23**	16	$20^{\triangle}$
Tokyo	$23^{\triangle}$	$24^{\triangle}$	**19**	$21^{\triangle}$	$26^{\triangle}$	**22**	16	$21^{\triangle}$
Sendai	$23^{\triangle}$	$25^{\triangle}$	$21^{\triangle}$	$22^{\triangle}$	25	**23**	$20^{\triangle}$	$19^{\triangle}$
Sapporo	$25^{\triangle}$	22	$22^{\triangle}$	20	25	**20**	$19^{\triangle}$	$22^{\triangle}$
Uniform	22		20		25		16	
