# Supplementary material for: Vulnerable connectivity caused by local communities in spatial networks
Source: PLoS One. 2025 Jul 2;20(7):e0327203. doi: 10.1371/journal.pone.0327203 (PMC12221043; doi:10.1371/journal.pone.0327203)
Supplement: S12 Table — Sparsity Index (SI(Gw)) of RNG and GG for different Japanese cities (N = 1024). Values are calculated based on normalized edge distances, representing the spatial sparsity of each configuration. Uniform and 2D lattice cases serve as comparative baselines for sparsity. 2D lattice havs the highest SI(Gw), which may result from long edges between faraway nodes. (PDF) [file pone.0327203.s050.pdf]

Vulnerable connectivity caused by local communities in spatial networks

Yingzhou MOU[1*] and Yukio HAYASHI[1]

[1]Japan Advanced Institute of Science and Technology, Nomi-city, Ishikawa 923-1292, Japan

* mouyingzhou@outlook.com

Abstract

Local communities by concentration of nodes connected with short links are widely observed in spatial networks. However, how such structure affects robustness of connectivity against malicious attacks remains unclear. This study investigates the impact of local communities on the robustness by modeling planar infrastructure networks whose node's locations are based on statistical population data. Our research reveals that the robustness is weakened by strong local communities in spatial networks. These results highlight the potential of long-distance links in mitigating the negative effects of local community on the robustness.

Table S12

Cities	RNG		GG	
	Inv.	Pop.	Inv.	Pop.
Fukuoka	0.4235	0.9489	0.4214	0.9437
Hiroshima	0.4163	0.9470	0.4099	0.9405
Keihan	0.4634	0.8842	0.4572	0.8406
Nagoya	0.4638	0.8651	0.4500	0.8254
Tokyo	0.4270	0.8825	0.4181	0.8652
Sendai	0.4660	0.9559	0.4594	0.9468
Sapporo	0.5217	0.9673	0.5139	0.9602
Uniform	0.3025		0.3001	
2D Lattice	0.9738		0.9200	
