# Supplementary material for: Vulnerable connectivity caused by local communities in spatial networks
Source: PLoS One. 2025 Jul 2;20(7):e0327203. doi: 10.1371/journal.pone.0327203 (PMC12221043; doi:10.1371/journal.pone.0327203)
Supplement: S13 Table — The Pearson’s correlation coefficients (r) and significance levels (p-values) between the sparsity index (SI) and robustness measures (R and qc) in RNG and GG against three attack strategies (RB, ID, and RF). Bold values indicate statistically significant results (p<0.05). A negative correlation (r<0) suggests that higher sparsity (larger SI) is associated with lower robustness, implying that spatially sparser networks are more vulnerable. Conversely, a positive correlation (r>0) indicates that sparsity is associated with higher robustness, which may occur in certain configurations where redundant links or grid-like structures compensates for sparsity. (PDF) [file pone.0327203.s051.pdf]

Vulnerable connectivity caused by local communities in spatial networks

Yingzhou MOU[1*] and Yukio HAYASHI[1]

[1]Japan Advanced Institute of Science and Technology, Nomi-city, Ishikawa 923-1292, Japan

* mouyingzhou@outlook.com

Abstract

Local communities by concentration of nodes connected with short links are widely observed in spatial networks. However, how such structure affects robustness of connectivity against malicious attacks remains unclear. This study investigates the impact of local communities on the robustness by modeling planar infrastructure networks whose node's locations are based on statistical population data. Our research reveals that the robustness is weakened by strong local communities in spatial networks. These results highlight the potential of long-distance links in mitigating the negative effects of local community on the robustness.

Table S13

**Measures**	**RB**		**ID**		**RF**	
	RNG	GG	RNG	GG	RNG	GG
**Pearson** r (R)	-0.4071	**-0.6514**	**+0.7017**	-0.3532	+0.0786	**-0.7300**
**Pearson** r (q_c)	**-0.5468**	-0.3181	+0.3498	**-0.5543**	-0.2504	-0.4458
p**-value** (R)	0.1486	**0.0116**	**0.0052**	0.2155	0.7894	**0.0030**
p**-value** (q_c)	**0.0430**	0.2678	0.2202	**0.0397**	0.3879	0.1101
