# Supplementary material for: Vulnerable connectivity caused by local communities in spatial networks
Source: PLoS One. 2025 Jul 2;20(7):e0327203. doi: 10.1371/journal.pone.0327203 (PMC12221043; doi:10.1371/journal.pone.0327203)
Supplement: S14 Table — The Pearson’s correlation coefficient (r) and significance level (p-value) between modularity Q and sparsity index SI(Gw) for RNG and GG. The correlation is statistically significant only for GG (p<0.05), indicating monotone increasing trends association between modularity Q and spatial sparsity index SI(Gw) in GG. (PDF) [file pone.0327203.s052.pdf]

Vulnerable connectivity caused by local communities in spatial networks

Yingzhou MOU[1*] and Yukio HAYASHI[1]

[1]Japan Advanced Institute of Science and Technology, Nomi-city, Ishikawa 923-1292, Japan

* mouyingzhou@outlook.com

Abstract

Local communities by concentration of nodes connected with short links are widely observed in spatial networks. However, how such structure affects robustness of connectivity against malicious attacks remains unclear. This study investigates the impact of local communities on the robustness by modeling planar infrastructure networks whose node's locations are based on statistical population data. Our research reveals that the robustness is weakened by strong local communities in spatial networks. These results highlight the potential of long-distance links in mitigating the negative effects of local community on the robustness.

Table S14

Metric	RNG	GG
r	0.2694	0.5930
p-value	0.3315	**0.0198**
